# A Supervised Fine-Tuned Large Language Model for Lifestyle Management in Patients With Prostate Cancer: Development and Evaluation Study

**DOI:** 10.2196/92663

**Published:** 2026-07-21

**Authors:** Fangyuan Jiang, Qiuwen Yang, Xin Zheng, Nan Yin, Jiayi Zhang, Tu Lan, Yuanjun Wu, Yuxin Lin, Kui Jiang, Yalan Chen

**Affiliations:** 1 Department of Medical Informatics, School of Medicine, Nantong University, Qixiu Road 19#, Nantong, Jiangsu, 226001, China, +86 0513 8505 1891; 2 Department of Urology, The First Affiliated Hospital of Soochow University, Suzhou, Jiangsu, China

**Keywords:** prostate cancer, lifestyle intervention, large language model, LLM-as-a-judge, retrieval-augmented generation, supervised fine-tuning, conversational AI, patient education

## Abstract

**Background:**

Lifestyle interventions for patients with prostate cancer have been shown to improve treatment adherence and quality of life. However, there remains a lack of large language models (LLMs) capable of delivering individualized and professional lifestyle recommendations under clearly defined medical safety boundaries and controlled evidence sources.

**Objective:**

This study aimed to develop and evaluate a supervised fine-tuned LLM—PCaPLMM_SFT (Prostate Cancer Patient Lifestyle Management Model via Supervised Fine-Tuning)—to support health literacy improvement and lifestyle self-management among patients with prostate cancer.

**Methods:**

We searched English-language literature primarily from PubMed (February 2015 to February 2025) to build a structured lifestyle management knowledge base covering diet, physical activity, weight management, medication adherence, and psychological support. We used a retrieval-augmented generation pipeline to generate patient-style question-answer (QA) pairs from retrieved knowledge slices. Bilingual English-Chinese QA data were generated from English-language source evidence through patient-oriented reformulation and retrieval-augmented generation–based answer generation, and independent English and Chinese test sets were constructed to assess bilingual QA performance. We trained Baichuan2-7B-Chat using a 2-stage strategy, consisting of continued pretraining, followed by supervised fine-tuning with low-rank adaptation. Model outputs were evaluated in 2 double-blind rounds by referee LLMs (Qwen3-Max and DeepSeek-R1) and compared with GPT-3.5-Turbo and the base Baichuan2-7B-Chat using 2500 queries across 5 lifestyle scenarios. Additionally, 3 domain experts conducted a blinded review of 50 QA samples (10 per scenario). We used the Mann-Whitney *U* test with effect size *r*, and Benjamini-Hochberg false discovery rate correction, and examined consistency using intraclass correlation coefficients.

**Results:**

Based on 2211 included publications, we constructed the PCaPLMM_SFT-Train dataset. The knowledge base yielded >150,000 structured knowledge slices. After 2 rounds of review, we obtained 42,330 single-turn QA pairs and 3008 multiturn dialogues, and the supervised fine-tuning phase used 45,338 structured QA samples. In the dual-round referee LLM assessment, PCaPLMM_SFT consistently outperformed Baichuan2-7B-Chat across dimensions and showed comparable or superior performance to GPT-3.5-Turbo across 5 lifestyle scenarios. Consistency analyses indicated moderate to good agreement between referee models across rounds, supporting the robustness of the comparative evaluation.

**Conclusions:**

PCaPLMM_SFT demonstrates the feasibility of constructing a medical lifestyle–focused LLM by integrating structured medical knowledge, QA-style training data, and a multilayer evaluation system. This framework provides a reproducible methodological foundation for evidence-based health education and lifestyle management and establishes groundwork for future evaluation in real-world health management settings.

## Introduction

Prostate cancer is the second most common malignancy among men worldwide, and its incidence and disease burden continue to rise. Evidence indicates that lifestyle factors can significantly influence the quality of life and disease trajectory for patients. These factors specifically include diet, weight management, and physical activity [1]. Multiple randomized controlled trials and real-world intervention studies have validated the safety and efficacy of such lifestyle interventions on clinical outcomes, while also highlighting the persistent demand from both patients and health care professionals for individualized, evidence-based lifestyle guidance [2-4].

With the rapid advancement of AI, large language models (LLMs) such as GPT-4 and BioRAG have shown strong potential in medical question answering, abstract generation, and clinical decision support [5,6]. Recent studies on LLM-based medical chatbots have further highlighted their potential in patient-facing health information delivery and communication, particularly in oncology-related patient education, while emphasizing risks related to hallucination, bias, privacy, and governance, as well as the need for curated knowledge sources, quality control, and continuous monitoring [7,8]. For example, GPT-4 achieved above-average human-level performance in the United States Medical Licensing Examination, drawing wide attention to its potential clinical applications. However, these general-purpose models face two critical challenges in real-world medical scenarios: (1) the lack of structured domain-specific knowledge, which often leads to incomplete or incorrect retrieval of relevant evidence [9]; and (2) factual inaccuracies or hallucinations, which raise concerns about their safe use in clinical communication and patient education [10].

Recent studies in critical care medicine have demonstrated that integrating domain knowledge bases (KBs) with AI can substantially improve prediction and decision support. For example, Yang et al [11] developed a transformer-based time series framework for intensive care unit patients with sepsis, achieving high predictive accuracy and interpretability by using longitudinal physiological data. Similarly, Zhang et al [12] proposed the MetaSepsisKnowHub platform, which combines retrieval-augmented generation (RAG) with curated biomarker knowledge to enhance LLM factual accuracy and clinical applicability. These efforts underscore the importance of domain-enhanced AI systems and provide valuable methodological inspiration. However, existing oncology-focused LLM systems and broader RAG-based medical LLM frameworks have mainly emphasized treatment-related information retrieval, radiotherapy education, molecular treatment recommendation, cancer progression prediction, or clinical decision support.

To our knowledge, no study has specifically developed and systematically evaluated an LLM fine-tuned for patient-facing lifestyle management in patients with prostate cancer. This task requires not only high comprehensibility and robust evidence alignment but also empathy and consistent safety safeguards in real-world dialogues. Authentic physician-patient conversation data are scarce. To address this challenge, it is essential to explore methods that generate high-quality, literature-driven training corpora and apply supervised fine-tuning (SFT) to enhance both the professionalism and the reliability of model outputs. Furthermore, Song et al [13] have shown that medical LLMs often suffer from hallucinations and limited domain grounding, and that incorporating structured, literature-anchored knowledge—particularly through knowledge graph–based retrieval augmentation—can markedly improve model accuracy, response consistency, and diagnostic reliability in complex clinical settings.

To address these unmet needs, we developed the PCaPLMM_SFT (Prostate Cancer Patient Lifestyle Management Model via Supervised Fine-Tuning), a domain-specific LLM built on the Baichuan2-7B-Chat architecture [14]. The model integrates literature-driven question-answer (QA) generation and supervised instruction tuning to build structured corpora across multiple lifestyle-related scenarios. Unlike prior oncology-focused or RAG-based medical LLM frameworks that mainly emphasize retrieval-grounded answering or clinical decision support, PCaPLMM_SFT focuses on patient-facing lifestyle management for patients with prostate cancer and uses RAG not only for evidence-grounded inference but also for transforming PubMed-based evidence into patient-style QA training data for domain-adaptive SFT.

Accordingly, this study aimed to construct and evaluate PCaPLMM_SFT as a proof-of-concept framework for AI-assisted lifestyle management in patients with prostate cancer. Specifically, we (1) built a structured, literature-driven QA corpus covering major lifestyle domains such as diet, exercise, weight management, medication adherence, and psychological support; (2) applied SFT to enhance the model’s domain comprehension, empathy, and factual reliability; and (3) established a standardized, multidimensional evaluation system to assess evidence alignment, comprehensibility, relevance, empathy, and feasibility.

Together, these objectives provide a reproducible pathway for building disease-specific LLMs and assessing their potential in patient education and chronic disease management. The framework integrates literature-to-QA automation and structured knowledge organization, enabling scalable, evidence-based model development.

## Methods

### Study Design

**Figure 1.**
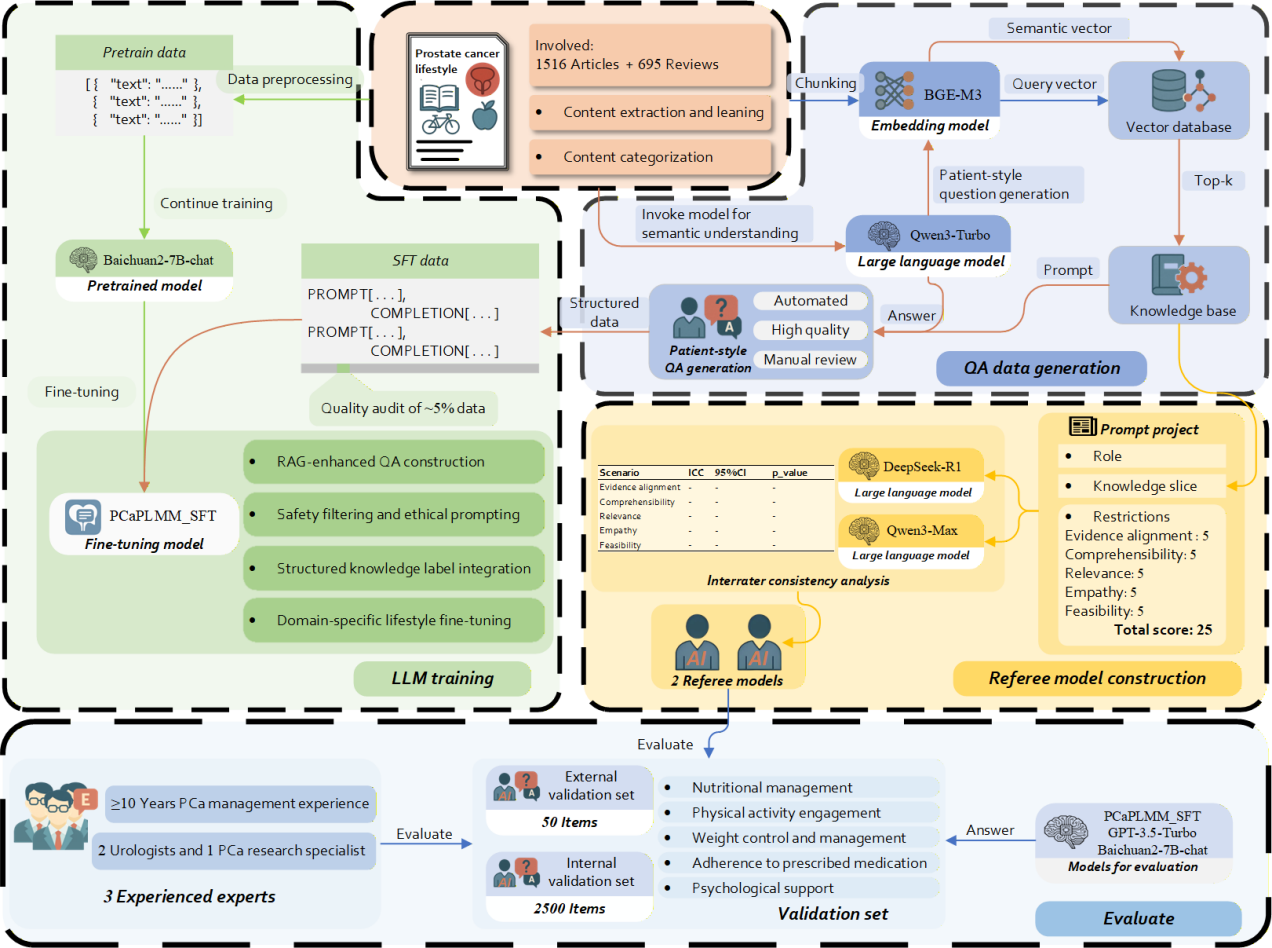
Development workflow of the PCaPLMM_SFT model. The workflow illustrates the end-to-end pipeline, encompassing literature retrieval, corpus cleaning and QA construction, model training, test-set validation, and iterative refinement based on evaluation feedback. BGE-M3: BAAI General Embedding M3; ICC: intraclass correlation coefficient; LLM: large language model; PCa: prostate cancer; PCaPLMM_SFT: Prostate Cancer Patient Lifestyle Management Model via Supervised Fine-Tuning; QA: question-answer; RAG: retrieval-augmented generation; SFT: supervised fine-tuning.

We followed a multistage systematic workflow to develop the PCaPLMM_SFT (Figure 1). The process included 5 major steps: data preparation, construction of QA datasets with RAG and LLMs, model training, referee model design, and model evaluation. It forms a closed-loop pipeline from knowledge extraction to model optimization and assessment.

### Data Preparation

The data were primarily obtained from English-language PubMed publications. Publications between February 2015 and February 2025 were retrieved using search terms related to prostate cancer and lifestyle management, including lifestyle, exercise, and diet. The full PubMed search strategy and eligibility criteria are provided in Multimedia Appendix 1. In addition to original research papers, secondary research, such as systematic reviews and meta-analyses, was also included to supplement real-world evidence gaps.

To meet LLMs’ training requirements, all raw documents underwent standardized preprocessing before inclusion. Through multiple rounds of cleaning and semantic extraction, irrelevant content—such as author information, correspondence addresses, DOIs, and copyright statements—was removed. The remaining content was transformed into JSON format suitable for LLM learning (eg, “text”: “paragraph content”). For complex review papers and documents with irregular structures, we applied a hybrid automated manual annotation strategy to maintain semantic consistency and ensure high data quality, providing a reliable foundation for subsequent QA construction.

### Construction of the Prostate Cancer Lifestyle KB

Cleaned texts were segmented into minimal semantic units, or chunks, based on paragraph boundaries, topic continuity, and medical semantic integrity rather than arbitrary fixed-length splitting. For each chunk, source metadata, including publication information, lifestyle domain, original section, and evidence source, were retained to ensure traceability. Duplicated, noninformative, or structurally abnormal chunks were removed through automated filtering and manual spot-checking. Each chunk was embedded into high-dimensional semantic vectors using the BGE-M3-embedding model and stored in a FAISS (Facebook Artificial Intelligence Similarity Search)–based vector database together with the original text and metadata. This process formed a structured KB enabling efficient and traceable semantic retrieval for downstream QA and inference tasks [15].

### Retrieval-Augmented Generation

During QA generation, query vectors were generated from predefined keywords or semantic seeds. Top-k semantic retrieval (k=20) was performed on the KB to obtain relevant knowledge slices. The value of k=20 was selected as a pragmatic balance between evidence coverage and noise control: a smaller k may miss complementary evidence, whereas a larger k may introduce weakly relevant fragments and distract the model from the most relevant information [16-18]. Retrieved content and preconstructed questions were combined and fed into an LLM via prompting templates, producing answers grounded in medical evidence. RAG was used both for (1) retrieving evidence to generate QA training data and (2) retrieving evidence at inference time to ground responses. Retrieved slices were ranked by similarity, and no additional fixed similarity threshold was applied before response generation. The current system did not implement a formal evidence-grade weighting algorithm or automated conflict adjudication mechanism for inconsistent retrieved slices. When retrieved evidence was heterogeneous, the model was guided by evidence-oriented prompts to generate cautious and individualized responses while avoiding absolute conclusions. This approach was intended to reduce hallucinations and support the medical accuracy and contextual coherence of the outputs.

### Patient-Oriented Question Reformulation

Based on the prostate cancer lifestyle knowledge slices, core factual questions were extracted through medical templates. These base questions were then reformulated by Qwen3-Turbo into more natural, conversational, and patient-oriented queries. To prevent redundancy and semantic conflicts, semantic similarity filtering was applied to remove duplicate or stylistically inconsistent queries, thereby improving diversity and consistency.

### High-Quality Answer Generation

Each patient-style question and its corresponding knowledge slice were combined using a unified prompt template and input into Qwen3-Turbo for answer generation. Because Qwen3-Turbo was used as the primary QA generator, the generated corpus may reflect model-specific biases in linguistic style, knowledge coverage, safety-related phrasing, and reasoning patterns. To reduce unconstrained model generation, answers were generated from retrieved literature-based knowledge slices rather than solely from the model’s parametric knowledge. The prompt required the model to prioritize evidence-based recommendations, precise terminology, and consistency with the retrieved medical evidence. The generated QA pairs were formatted in ShareGPT JSON style to ensure compatibility for SFT.

To ensure data quality, a manual and automated quality control process was applied. We manually reviewed generated QA pairs using a stratified sampling strategy; this manual review was designed as a stratified quality control audit rather than a complete manual validation of the entire corpus. The review was conducted independently by 2 researchers (XW and HH) with medical backgrounds. Manual assessment evaluated factual accuracy, evidence alignment, safety, linguistic clarity, structural validity, and actionability. This audit was used to identify common quality problems, refine editing criteria, and guide subsequent corpus cleaning and standardization. Interrater agreement metrics were calculated and are reported in Multimedia Appendix 2. Discrepant samples were further reviewed and used to guide correction, revision, or exclusion. This process was supplemented by automated checks, including semantic deduplication, terminology filtering, format validation, and logical conflict detection. Together, these procedures removed misleading, ungrammatical, or structurally abnormal samples, and improved the professionalism, naturalness, and standardization of the dataset [19].

### Group-Based Semantic Partitioning to Prevent Train-Evaluation Contamination

To prevent train-test leakage, we adopted a base-question-group splitting strategy. Each base question and its 1‐2 paraphrased variants were grouped as a single unit and assigned entirely to either the training or evaluation set to avoid semantic overlap across datasets. In addition, multilevel deduplication and semantic similarity filtering were applied. All queries were encoded using BGE-M3, and cosine similarity between training and evaluation sets was computed. Cross-set query pairs with high similarity (near-paraphrase cases) were automatically removed, ensuring strict semantic isolation between training and evaluation data.

### Task Definition

To clarify the functional scope of the QA workflow, the QA task was defined as a composite generative process performed autonomously by PCaPLMM_SFT after continued pretraining and SFT. The task integrated two capabilities: (1) medical knowledge–based reasoning and (2) generation of patient-oriented lifestyle recommendations. Inputs included a fixed system prompt (eg, “You are PCaPLMM_SFT...provide professional and evidence-based responses”), brief patient information, and a lifestyle-related query. The model first performed medical semantic interpretation and evidence-based reasoning using the structured literature KB and then converted the inferred content into clear, empathetic, and actionable recommendations. Outputs were restricted to a single evidence-grounded and medically accurate answer. Task performance was evaluated across the 5 dimensions: evidence alignment, comprehensibility, relevance, empathy, and feasibility.

### Training Strategy

This study adopted a 2-stage training strategy for PCaPLMM_SFT. Stage 1 performed parameter-efficient continued pretraining using low-rank adaptation adapters on the Baichuan2-7B-Chat backbone. Training was conducted in LLaMA-Factory with a 2048-token context window, the AdamW optimizer (learning rate 2×10^–^⁴), cosine scheduling with 200 warm-up steps, weight decay, gradient clipping (maximum norm=1.0), a per-device batch size of 4, and 8 gradient accumulation steps for 3 epochs. Stage 2 fine-tuned the stage 1 checkpoint using domain-adaptive supervised fine-tuning with low-rank adaptation under a 1024-token context window, AdamW (2×10^–^⁵), and a fixed 3-epoch schedule.

### Referee Model Construction

The referee models were implemented by accessing the application programming interfaces of DeepSeek-R1 and Qwen3-Max through an open model service platform. Using prompt engineering, we constructed structured, outline-based scoring instructions, forming an LLM-based referee mechanism. This LLM-as-a-judge framework was adopted based on prior studies showing that large LLMs can generate structured evaluation signals under standardized rubrics, although results may be affected by prompt sensitivity and model-specific preferences [20-22]. The evaluation prompt templates incorporated knowledge slices, role definitions, scoring criteria, and explicit restrictions (Multimedia Appendix 3) to support a consistent evaluation context and produce reproducible assessment outputs. To examine the robustness of the referee LLM outputs, we further assessed their interround stability, intermodel consistency, and human-LLM consistency.

### Multidimensional Scoring System

The referee mechanism assessed each candidate answer across 5 dimensions—evidence alignment, comprehensibility, relevance, empathy, and feasibility—using the detailed definitions and scoring criteria (Multimedia Appendix 4). A predefined safety rule was also applied to flag responses with potential patient safety risks. Because PCaPLMM_SFT was designed for patient education and lifestyle guidance rather than for diagnosis or treatment decision-making, unsafe outputs were operationalized using a threshold for potential patient harm: responses were considered unsafe if they could reasonably mislead patients toward harmful self-management behaviors or unsupervised changes in medical care. This predefined safety rule was used only for post hoc evaluation and not as a trigger for inserting disclaimers during generation. Unsafe labels were used for analysis rather than exclusion; flagged responses remained in the evaluation denominator for error analysis and reporting of representative failure cases. Responses were labeled “unsafe” when (1) the referee assigned a score of 1 on any subdimension of evidence alignment, indicating inconsistency with established findings; (2) the recommendation carried potential for direct patient harm, such as inappropriate medication suggestions, unsafe or contraindicated lifestyle or exercise advice, or omission of essential safety precautions; or (3) the answer encouraged patients to initiate, discontinue, or modify medical treatment without clinician supervision.

### Evaluation Procedure

A total of 2500 patient-style queries from English and Chinese test sets covering the 5 lifestyle management scenarios were used for evaluation. Candidate answers from all models were scored independently and blindly by the referee model in 2 rounds. Each dimension was rated on a 5-point Likert scale, ranging from “strongly disagree” (1) to “strongly agree” (5) (Multimedia Appendix 4).

### Referee Consistency Analysis

To evaluate the stability of scores at the individual dimension level, each “question× dimension” pair was treated as an independent scoring unit. Intraclass correlation coefficient (ICC) (3,k) was computed using a 2-way mixed-effects model (consistency and average measures). Additionally, Pearson and Spearman correlations were calculated based on a concatenated score vector stacking the 5-dimension–level scores for each question. This approach measures consistency on both continuous and ordinal scales.

### Human Expert Evaluation

To further validate the reliability of the referee LLM evaluations, we conducted a small-scale manual assessment by human experts. Three experts with ≥10 years of experience in prostate cancer management (2 urologists and 1 prostate cancer research specialist) independently evaluated the model-generated responses. A total of 50 QA samples were randomly selected, covering 5 core lifestyle scenarios (10 samples per scenario), based on prior studies [23,24]. This expert assessment served as a supplementary validation component to examine whether automated evaluation signals were broadly aligned with clinical judgment across key dimensions such as evidence alignment, safety, usefulness, feasibility, and patient-centered communication [21,25]. All texts were fully deidentified, and experts received only the QA pairs without any model identifiers to minimize potential evaluation bias. The expert assessment criteria, scoring dimensions, and consistency evaluation methods were kept consistent with those used in the referee LLM evaluation. The sample expert evaluation form for assessing multiple model outputs on lifestyle management in patients with prostate cancer is provided in Multimedia Appendix 5.

### Statistical Analysis

All statistical analyses were performed using R (version 4.3.1; R Foundation for Statistical Computing) and Python (version 3.10; Python Software Foundation) with the SciPy (version 1.11; SciPy Community), pandas, and numpy libraries. Referee evaluation scores of candidate models were compared using the Mann-Whitney *U* test for 2 independent groups. In addition to the *U* statistic and 2-sided *P* values, standardized *Z* scores and effect sizes (*r*) were calculated, where $r\text{=}\text{|}Z\text{|}/\sqrt{N}$ and *N* is the total sample size. Effect size interpretation followed Cohen thresholds: 0.1=small, 0.3=medium, and 0.5=large. The descriptive statistics (mean, SD) were summarized for each group, along with the corresponding 95% CIs. All multiple comparisons were corrected for the false discovery rate using the Benjamini-Hochberg procedure. A 2-sided *P*<.05 was considered statistically significant.

### Ethical Considerations

This study did not involve patient recruitment, clinical intervention, or the collection or processing of patient-level identifiable information and therefore did not constitute a clinical trial. The study protocol, including the expert evaluation process, was reviewed and approved by the Medical Ethics Committee of Nantong University, with the approval number 2025‐25. The evaluation was conducted by invited domain experts, who were informed of the study purpose, evaluation procedures, and intended use of the results before participation. All experts participated voluntarily and provided informed consent. The evaluation results are reported only in aggregate form, and no personally identifiable information is presented in the manuscript.

## Results

### PCaPLMM_SFT-Train Dataset

**Figure 2.**
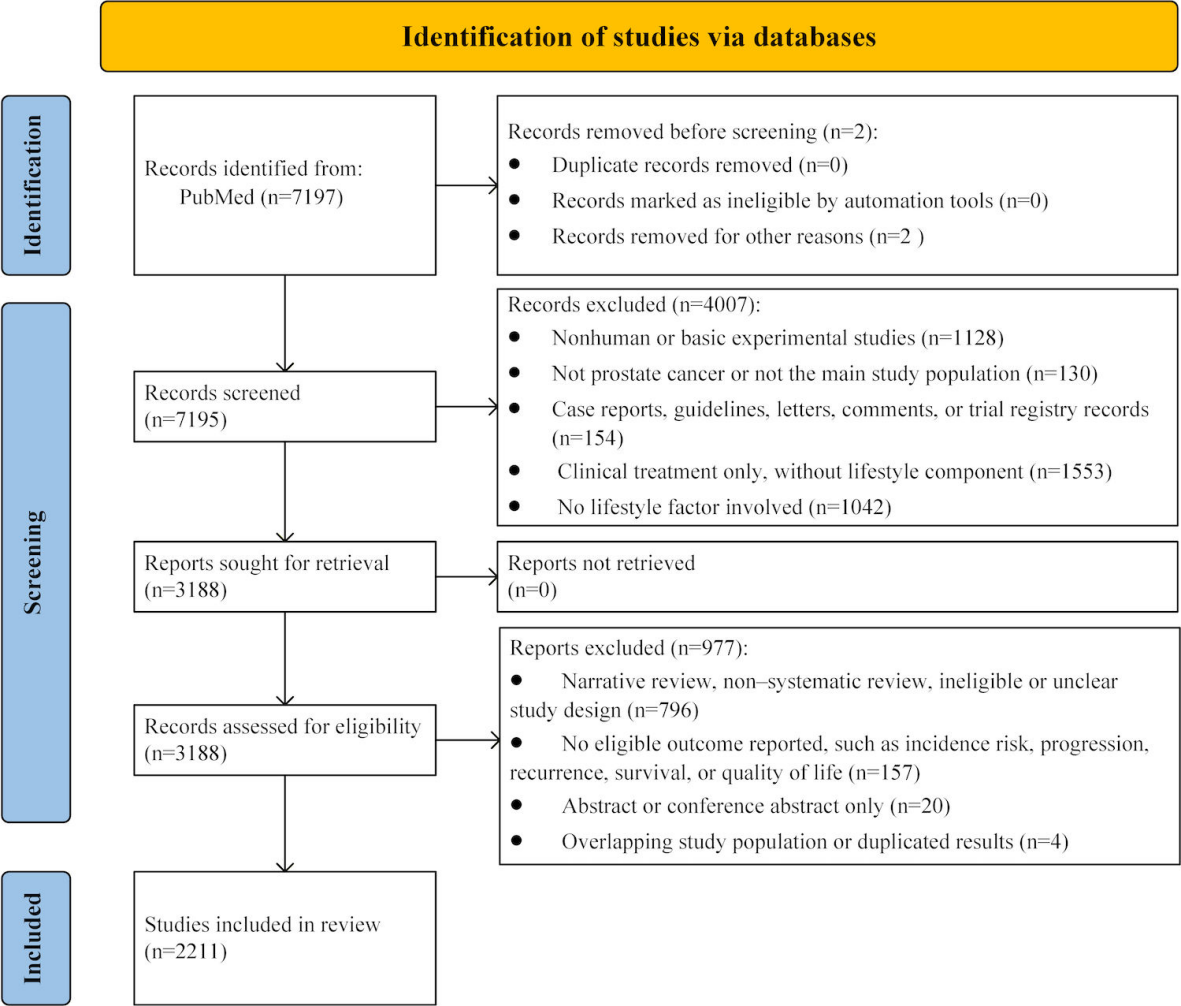
PRISMA (Preferred Reporting Items for Systematic Reviews and Meta-Analyses) flowchart.

Briefly, 7197 records were identified from PubMed, 7195 records were screened, 3188 reports were assessed for eligibility, and 2211 studies were finally included for KB construction. The PRISMA (Preferred Reporting Items for Systematic Reviews and Meta-Analyses) flow diagram of our review is shown in Figure 2.

Based on 2211 included publications, we constructed the PCaPLMM_SFT-Train dataset tailored to prostate cancer lifestyle management scenarios (Table 1). The dataset comprised 1516 original studies and 695 secondary research papers. The content covered 5 core themes: diet and nutrition, physical activity, weight management, psychological support, and medication adherence. After automated cleaning and manual review, irrelevant content and potentially sensitive information were removed. The texts were then structured and labeled by theme to form the foundational corpus.

**Table 1.** Composition of the PCaPLMM_SFT^a^-Train dataset.

Dataset name	Type	Volume	Description
Pretraining dataset	Text data	45,084 entries	Included 1516 original studies and 695 reviews for continued pretraining.
Single-turn dataset	Medical dialogues	42,330 pairs	Patient-style QA^b^ pairs covering diet, exercise, weight management, psychological support, and medication adherence.
Multiturn dataset	Multiturn dialogues	3008 pairs	Extended multiturn interactions simulating lifestyle management scenarios.

a PCaPLMM_SFT: Prostate Cancer Patient Lifestyle Management Model via Supervised Fine-Tuning.

b QA: question-answer.

Within the RAG framework, we used Qwen3-Turbo to automatically generate QA pairs. Questions were produced through template-based extraction, followed by paraphrasing to simulate patient-style queries. Answers were generated from knowledge slices retrieved from the structured KB and were manually screened to ensure accuracy and consistency. To further enhance bilingual understanding and response generation, we constructed a bilingual English-Chinese patient-style QA corpus from the same English-language source evidence through patient-oriented reformulation and RAG-based answer generation. Although non–English literature databases were not independently searched, the generated corpus encompassed a broad range of lifestyle-related domains and incorporated culturally and regionally specific expressions. After 2 rounds of review, we obtained 42,330 single-turn QA pairs and 3008 multiturn dialogues.

### Automated QA Generation System

**Figure 3.**
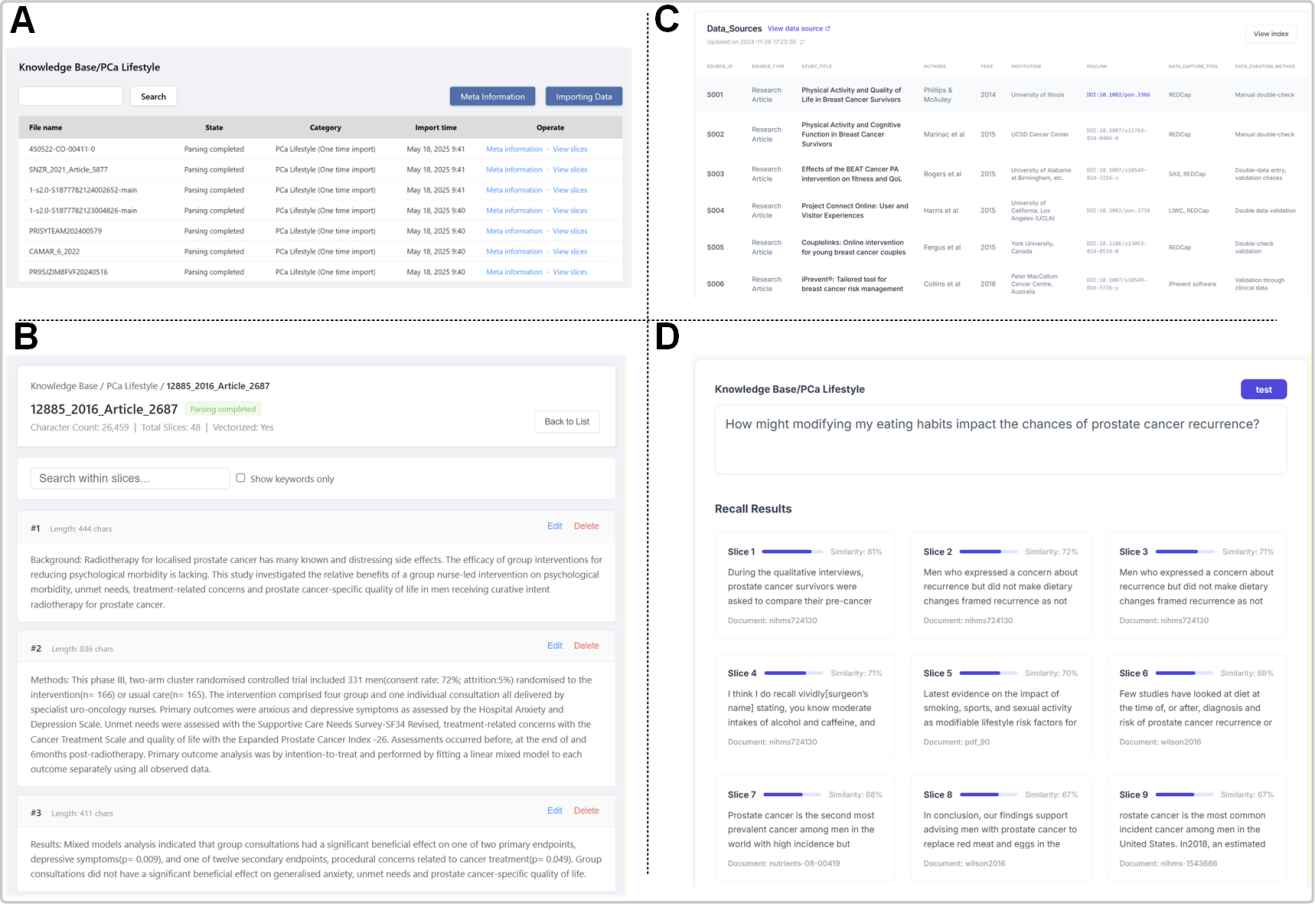
Interface of the PCaPLMM_SFT knowledge base. This figure illustrates the core components of the knowledge base: (A) import and management, (B) semantic slicing, (C) structured indexing, and (D) similarity-based retrieval. PCa: prostate cancer; REDCap: Research Electronic Data Capture; SAS: Statistical Analysis System.

The QA generation system integrated structured medical literature knowledge with semantic retrieval and LLM-based generation to produce high-quality, automated patient-style QA content. The KB was constructed by importing unstructured medical texts, segmenting them into semantic vectors, and extracting metadata. A total of more than 150,000 knowledge slices were generated, covering all major lifestyle-related conversational scenarios. Figure 3 illustrates the KB management interface, which includes literature import (Figure 3A), content editing (Figure 3B), structured indexing (Figure 3C), and semantic debugging modules (Figure 3D). Qwen3-Turbo was used to automatically generate patient-style questions, achieving interactive linkage between knowledge construction and data generation.

For each knowledge slice, the system generated 5 base questions aligned with lifestyle contexts. Each question was then paraphrased 1‐2 times by an LLM to enrich the linguistic diversity while preserving its core medical meaning. All questions were documented with their contents, scenario, generation type (original or paraphrased), and source reference. Examples are presented in Table 2.

**Table 2.** Examples of patient-style question generation.

Question	Scenario	Type	Source file
What dietary changes should I make to potentially improve my prostate cancer outcome?	Diet	Original	1-s2.0-S0013935122007435-main.pdf
What modifications to my diet might enhance the prognosis of my prostate cancer?	Diet	Paraphrased	1-s2.0-S0013935122007435-main.pdf
Which dietary adjustments could potentially improve the results of my prostate cancer treatment?	Diet	Paraphrased	1-s2.0-S0013935122007435-main.pdf
Would regular aerobic exercise help manage symptoms and improve my quality of life after a prostate cancer diagnosis?	Exercise	Original	1-s2.0-S0013935122019193-main.pdf
Can consistent aerobic exercise alleviate symptoms and enhance my quality of life following a prostate cancer diagnosis?	Exercise	Paraphrased	1-s2.0-S0013935122019193-main.pdf
Is engaging in regular aerobic exercise beneficial for controlling symptoms and boosting my quality of life post–prostate cancer diagnosis?	Exercise	Paraphrased	1-s2.0-S0013935122019193-main.pdf
Would increasing my physical activity improve my psychological well-being during prostate cancer treatment?	Psychological support	Original	12874_2021_Article_1486.pdf
Can engaging more in physical activities enhance my mental health while receiving prostate cancer treatment?	Psychological support	Paraphrased	12874_2021_Article_1486.pdf
Is boosting my level of exercise likely to positively impact my psychological state during prostate cancer therapy?	Psychological support	Paraphrased	12874_2021_Article_1486.pdf

**Figure 4.**
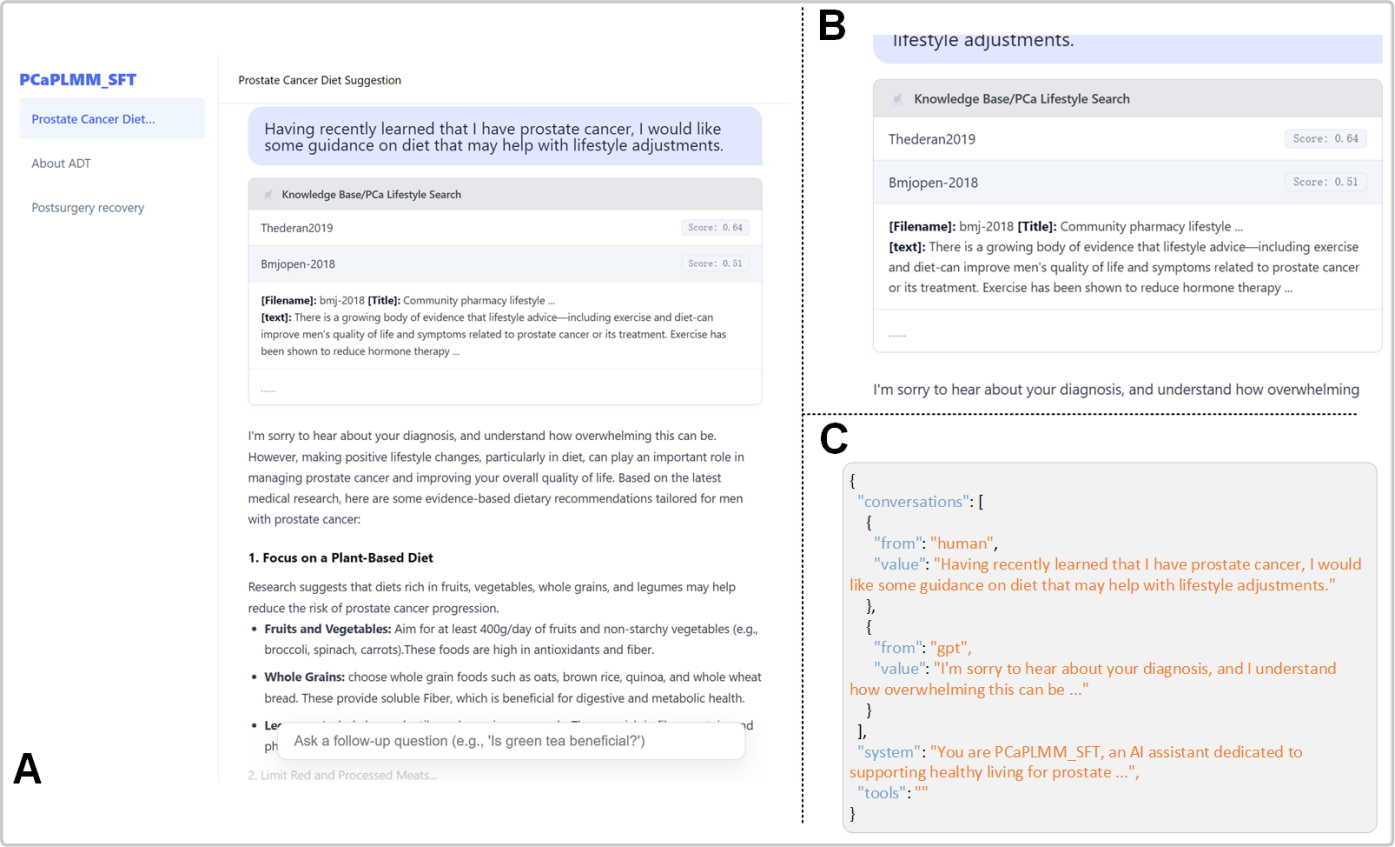
Example of knowledge-based question-answer (QA) generation. This figure shows the structured QA pair construction: (A) natural language input and retrieval, (B) extracted literature fragments, and (C) structured model output presented in the ShareGPT format. ADT: androgen deprivation therapy; PCa: prostate cancer; PCaPLMM_SFT: Prostate Cancer Patient Lifestyle Management Model via Supervised Fine-Tuning.

Following knowledge construction and question generation, we produced QA pairs that were explicitly grounded in medical evidence. For instance (Figure 4A), given the query “Having recently learned that I have prostate cancer, I would like some guidance on diet that may help with lifestyle adjustments,” the system retrieved 7 relevant research fragments (Figure 4B). The generated answer integrated dietary recommendations such as plant-based diets, reduced red meat and sugar intake, and appropriate fat consumption. The outputs were professional, structured, and standardized in ShareGPT JSON format (Figure 4C). To quantify dataset quality, we manually reviewed a stratified sample of 2400 QA pairs, representing 5.62% (2400/42,705) of the prefinal QA candidate corpus used for corpus auditing. Each sample was categorized by required editing level (Multimedia Appendix 6): 50.71% (1217/2400) needed only minor phrasing or structural refinements, 24.58% (590/2400) required major revisions due to factual or evidence misalignment, 22.79% (547/2400) were accepted without changes, and 1.92% (46/2400) were discarded for severe or noncorrectable errors. The major revision rate reflects QA quality and not the residual error in the final SFT dataset. Samples requiring major revision were revised according to retrieved evidence, while noncorrectable or potentially misleading samples were removed. The validated editing criteria derived from this audit were then applied to clean the remaining dataset. Representative failure cases are provided in Multimedia Appendix 7.

### Model Training of PCaPLMM_SFT

**Figure 5.**
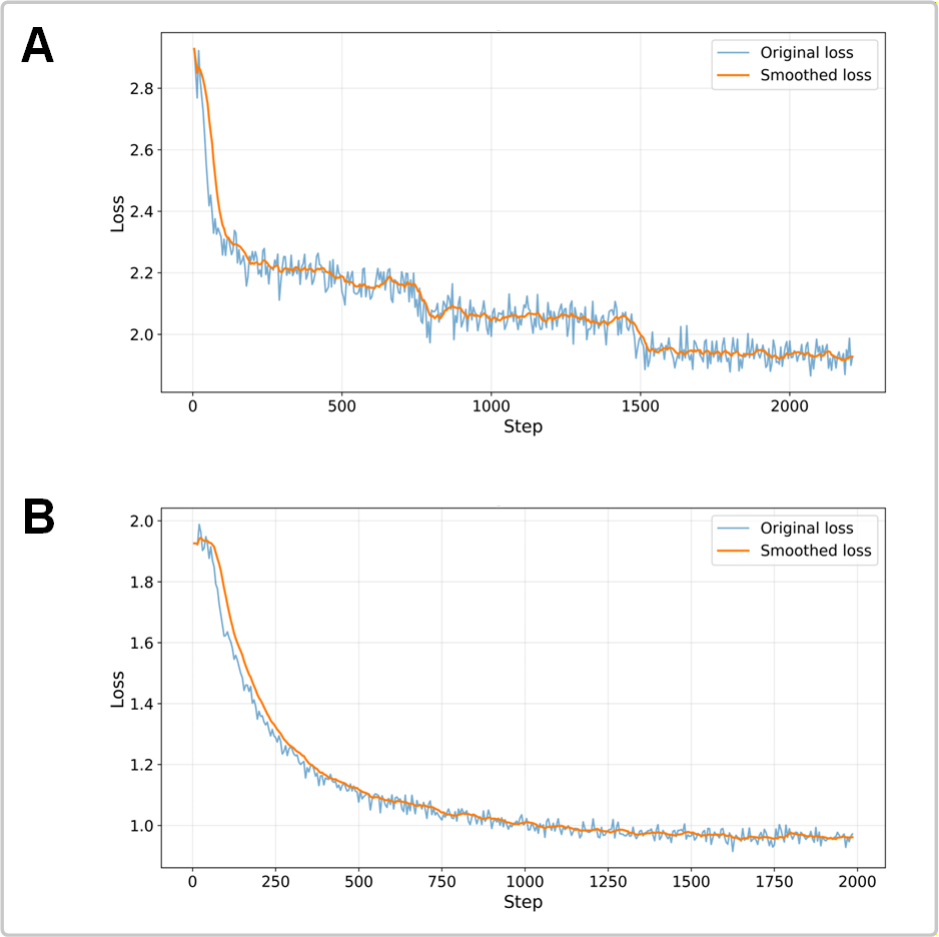
Training loss curves of PCaPLMM_SFT (Prostate Cancer Patient Lifestyle Management Model via Supervised Fine-Tuning). The curves display loss trajectories across (A) the continued pretraining phase and (B) the supervised fine-tuning phase.

The continued pretraining phase consisted of more than 2200 training steps. The loss decreased from approximately 2.9 to below 1.8 and stabilized after approximately 1600 steps (Figure 5A). A total of 23,263,936 tokens were used during this phase. In the subsequent SFT phase, 45,338 structured QA samples were used, with a total training volume of 60,788,864 tokens. The loss declined from approximately 2.0 to approximately 0.9 and converged after approximately 1750 steps (Figure 5B). The final model weights and configuration files have been publicly released on the Hugging Face platform (see the Data Availability section).

### Referee Model Performance and Error Analysis

Across both evaluation rounds, PCaPLMM_SFT consistently outperformed Baichuan2-7B-Chat across all dimensions and surpassed GPT-3.5-Turbo in most metrics (Table 3). Under Qwen3-Max, PCaPLMM_SFT achieved evidence alignment mean scores of 4.156 (SD 0.591) and 4.155 (SD 0.591), significantly higher than Baichuan2-7B-Chat (|*r*|=0.601‐0.614; *P*<.001) and GPT-3.5-Turbo (|*r*|=0.299‐0.305; *P*<.001). The model also showed clear advantages in feasibility dimension (mean 3.191, SD 0.717; mean 3.202, SD 0.706), again outperforming both baselines (*P*<.001).

**Table 3.** Referee evaluation scores for candidate models^a^.

Dimension (round)	PCaPLMM_SFT^b^, mean (SD), 95% CI	Baichuan2-7B-Chat, mean (SD), 95% CI	GPT-3.5-Turbo, mean (SD), 95% CI	*P*_1_ value (FDR^c^)	|*r*_1_|	*P*_2_ value (FDR)	|*r*_2_|
Qwen3-Max							
Evidence alignment ①	4.156 (0.591), 4.132-4.179	3.363 (0.906), 3.327-3.398	3.930 (0.522), 3.910-3.951	<.001	0.614	<.001	0.299
②	4.155 (0.591), 4.132-4.178	3.376 (0.902), 3.340-3.411	3.924 (0.518), 3.904-3.944	<.001	0.601	<.001	0.305
Comprehensibility ①	4.561 (0.437), 4.544-4.578	3.857 (0.857), 3.824-3.891	4.460 (0.368), 4.445-4.474	<.001	0.607	<.001	0.185
②	4.571 (0.417), 4.555-4.588	3.855 (0.857), 3.821-3.889	4.456 (0.365), 4.442-4.471	<.001	0.607	<.001	0.221
Relevance ①	2.899 (0.458), 2.881-2.917	1.959 (0.478), 1.940-1.978	2.832 (0.426), 2.815-2.849	<.001	0.821	<.001	0.109
②	2.911 (0.440), 2.894-2.928	1.962 (0.480), 1.943-1.981	2.829 (0.425), 2.812-2.846	<.001	0.823	<.001	0.148
Empathy ①	4.563 (0.474), 4.544-4.581	4.116 (0.862), 4.082-4.150	4.580 (0.363), 4.565-4.594	<.001	0.424	.57	0.012
②	4.576 (0.447), 4.558-4.593	4.107 (0.864), 4.073-4.141	4.570 (0.366), 4.555-4.584	<.001	0.44	.14	0.031
Feasibility ①	3.191 (0.717), 3.163-3.219	2.708 (0.707), 2.680-2.736	2.978 (0.544), 2.957-2.999	<.001	0.454	<.001	0.241
②	3.202 (0.706), 3.174-3.230	2.703 (0.711), 2.675-2.731	2.969 (0.552), 2.947-2.990	<.001	0.462	<.001	0.273
DeepSeek-R1							
Evidence alignment ①	4.345 (0.501), 4.325-4.365	3.740 (1.114), 3.696-3.783	3.915 (0.737), 3.886-3.944	<.001	0.491	<.001	0.446
②	4.310 (0.629), 4.285-4.335	3.544 (1.032), 3.504-3.584	3.885 (0.732), 3.857-3.914	<.001	0.663	<.001	0.428
Comprehensibility ①	4.657 (0.468), 4.639-4.675	4.350 (0.851), 4.317-4.383	4.607 (0.456), 4.589-4.625	<.001	0.331	<.001	0.079
②	4.624 (0.570), 4.602-4.646	4.321 (0.884), 4.287-4.356	4.626 (0.437), 4.609-4.643	<.001	0.608	.80	0.005
Relevance ①	3.739 (0.807), 3.707-3.770	3.000 (0.741), 2.971-3.029	3.260 (0.545), 3.239-3.282	<.001	0.578	<.001	0.422
②	3.242 (0.594), 3.219-3.266	2.993 (0.755), 2.963-3.023	3.259 (0.536), 3.238-3.280	<.001	0.851	.46	0.019
Empathy ①	4.634 (0.491), 4.615-4.654	4.463 (0.686), 4.436-4.490	4.698 (0.401), 4.683-4.714	<.001	0.219	<.001	0.095
②	4.637 (0.547), 4.615-4.658	4.431 (0.736), 4.402-4.460	4.703 (0.383), 4.688-4.718	<.001	0.462	<.001	0.101
Feasibility ①	3.968 (0.706), 3.940-3.995	3.392 (0.846), 3.358-3.425	3.373 (0.621), 3.348-3.397	<.001	0.479	<.001	0.53
②	3.741 (0.649), 3.715-3.766	3.283 (0.806), 3.251-3.314	3.298 (0.584), 3.275-3.321	<.001	0.737	<.001	0.483

a *P*_1_ and |*r*_1_| represent the comparative results between PCaPLMM_SFT and Baichuan2-7B-Chat, whereas *P*_2_ and |*r*_2_| correspond to the comparison between PCaPLMM_SFT and GPT-3.5-Turbo. *P* values are derived from 2-sided Mann-Whitney *U* tests and adjusted using the Benjamini-Hochberg procedure to control the false discovery rate. |*r*| denotes the absolute effect size, calculated from the standardized *Z* statistic and accompanied by 95% CIs estimated via Fisher *Z* transformation.

b PCaPLMM_SFT: Prostate Cancer Patient Lifestyle Management Model via Supervised Fine-Tuning.

c FDR: false discovery rate.

DeepSeek-R1 produced consistent findings: PCaPLMM_SFT showed significant gains in evidence alignment (mean 4.345, SD 0.501; mean 4.310, SD 0.629; *P*<.001) and delivered top performance in comprehensibility (4.624‐4.657) and empathy (4.634‐4.637), with significant margins over Baichuan2-7B-Chat (|*r*|≥0.219; *P*<.001).

Across the 2 referee LLMs, interrater and interround consistency analyses showed overall moderate to good agreement (Table 4). In round 1, Qwen3-Max and DeepSeek-R1 demonstrated moderate interrater consistency (ICC(3,k)=0.540; Pearson *r*=0.382; Spearman *ρ*=0.415), indicating generally comparable scoring patterns despite differences in scoring sensitivity. In round 2, interrater agreement was higher than in the first round (ICC(3,k)=0.747; Pearson *r*=0.600; Spearman *ρ*=0.655), reflecting a consistent scoring tendency between the 2 models when independently evaluating the same QA scenarios.

**Table 4.** Interrater and interround consistency of large language model referee evaluations^a^.

Comparison and metric type	Estimate	95% CI	*P* value
Qwen versus DeepSeek (round 1)			
ICC^b^(3,k)	0.540	0.530-0.550	<.001
Pearson *r*	0.382	—^c^	<.001
Spearman *ρ*	0.415	—	<.001
Qwen versus DeepSeek (round 2)			
ICC(3,k)	0.747	0.740-0.750	<.001
Pearson *r*	0.600	—	<.001
Spearman *ρ*	0.655	—	<.001
Round 1 versus round 2 (Qwen only)			
ICC(3,k)	0.816	0.810-0.820	<.001
Pearson *r*	0.690	—	<.001
Spearman *ρ*	0.719	—	<.001
Round 1 versus round 2 (DeepSeek only)			
ICC(3,k)	0.507	0.490-0.520	<.001
Pearson *r*	0.343	—	<.001
Spearman *ρ*	0.378	—	<.001

a ICC(3,k) was calculated using a 2-way mixed-effects model (consistency type and average measures) under the assumption of fixed raters. Pearson *r* and Spearman *ρ* were computed using a concatenated scoring vector formed by stacking the 5 evaluation dimensions for each question, allowing assessment of cross-dimensional agreement.

b ICC: intraclass correlation coefficient.

c Not available.

Within-model interround consistency showed clear differences: Qwen3-Max exhibited high reproducibility (ICC(3,k)=0.816; Pearson *r*=0.690; Spearman *ρ*=0.719), whereas DeepSeek-R1 demonstrated only moderate stability (ICC(3,k)=0.507; Pearson *r*=0.343; Spearman *ρ*=0.378). Overall, although the 2 referee models differed in scoring variability, their scoring trends remained reasonably aligned, supporting the robustness of the comparative evaluation across repeated assessments.

Across 2 independent generations, low-scoring outputs were grouped into 4 error categories (Table 5). PCaPLMM_SFT showed the lowest error rates overall, with evidence misalignment: 3.68%, overly general or insufficiently actionable advice: 9.28%, imprecise or insufficiently patient-centered language: 5.68%, and hallucinations: 5.32%. Baichuan2-7B-Chat demonstrated substantially higher error rates in all categories, particularly evidence misalignment (20.66%) and hallucinations (12.88%). GPT-3.5-Turbo showed intermediate performance, with a notably low rate of patient-centeredness errors (1.42%) (Multimedia Appendix 8). Evidence misalignment and hallucinations were treated as potentially risky error categories because they may mislead patients or provide unsupported lifestyle recommendations, although actual patient harm could not be directly assessed in this offline evaluation without real patient interactions. Overall, PCaPLMM_SFT presented fewer potentially risky errors, suggesting better evidence alignment and reliability in generating lifestyle recommendations.

**Table 5.** Representative failure cases of PCaPLMM_SFT^a^.

Error type	Example question	Original answer	Problem identified
Evidence misalignment	How much physical activity should I engage in weekly to support my prostate cancer treatment?	“You should perform at least 150 minutes of moderate-intensity or 75 minutes of vigorous-intensity aerobic activity per week, plus 2‐3 sessions of resistance training...androgen deprivation therapy may increase fat mass and decrease muscle mass, so exercise is helpful...”	The model incorrectly applied general population World Health Organization aerobic thresholds to a prostate cancer context. Evidence shows that men undergoing treatment—especially those on androgen deprivation therapy—benefit more from resistance-focused programs with individualized aerobic activity. Thus, the original answer overstated the suitability of population-level recommendations and overlooked cancer-specific safety and personalized exercise needs.
Insufficiently actionable advice	“What should I eat after prostate cancer surgery?”	“Maintain a balanced diet, eat vegetables and fruits, avoid fatty foods.”	Overall, lacks actionable details such as frequency, quantities, and specific limits.
Imprecise or non–patient-centered expression	“I feel anxious at night after surgery. What should I do?”	“Postoperative anxiety is a common psychiatric reaction. You need to improve your mood response.”	Language is technical and non–patient-centered; lacks reassurance and evidence-based coping strategies.
Hallucinations	How does maintaining a balanced diet rich in fruits and vegetables affect prostate cancer progression?	“A systematic review and meta-analysis. Prostate Cancer and Prostatic Diseases, 2016. 17(4): 357‐68.”	The model did not answer the question and instead produced a fabricated or irrelevant citation. This represents a hallucination where the model outputs a pseudoreference unrelated to the user query and fails to provide evidence-based dietary guidance. Such hallucinations may mislead readers by mimicking academic citations without substantive content.

a PCaPLMM_SFT: Prostate Cancer Patient Lifestyle Management Model via Supervised Fine-Tuning.

### Human Expert Ratings and Consistency With Referee LLMs

A total of 3 experts independently evaluated 50 deidentified QA samples, and their ratings were compared with those of the 2 LLM referee models (Table 6). Interexpert agreement was moderate, with ICC(3,k) ranging from 0.332 to 0.431 across expert pairs, and corresponding Pearson correlations of 0.394‐0.471 and Spearman correlations of 0.380‐0.511.

**Table 6.** Interrater reliability between human experts and LLM^a^ referee models.

Comparison and metric type	Estimate	95% CI	*P* value
Expert 1 and Expert 2			
ICC^b^(3,k)	0.413	0.360-0.460	<.001
Pearson *r*	0.471	—^c^	<.001
Spearman *ρ*	0.511	—	<.001
Expert 1 and Expert 3			
ICC(3,k)	0.332	0.270-0.390	<.001
Pearson *r*	0.394	—	<.001
Spearman *ρ*	0.380	—	<.001
Expert 2 and Expert 3			
ICC(3,k)	0.431	0.380-0.480	<.001
Pearson *r*	0.458	—	<.001
Spearman *ρ*	0.427	—	<.001
Qwen versus DeepSeek			
ICC(3,k)	0.689	0.660-0.710	<.001
Pearson *r*	0.527	—	<.001
Spearman *ρ*	0.506	—	<.001
Human Experts versus LLM Referees			
ICC(3,k)	0.474	0.410-0.530	<.001
Pearson *r*	0.367	—	<.001
Spearman *ρ*	0.365	—	<.001

a LLM: large language model.

b ICC: intraclass correlation coefficient.

c Not available.

The 2 referee LLMs (Qwen3-Max vs DeepSeek-R1) demonstrated higher intermodel agreement (ICC(3,k)=0.689; Pearson *r*=0.527; Spearman *ρ*=0.506), indicating a relatively consistent scoring pattern between models. When comparing human experts with LLM referees, consistency was moderate (ICC(3,k)=0.474, 95% CI 0.410‐0.530), with Pearson *r*=0.367 and Spearman *ρ*=0.365. These results suggest that although humans and LLM referees share similar overall ranking tendencies across QA outputs, divergence remains in fine-grained scoring decisions. Human experts’ evaluation scores for the candidate models are provided in Multimedia Appendix 9.

### Use Case Scenarios

We selected 5 common lifestyle management themes—diet, exercise, weight management, medication adherence, and psychological support—to construct the representative patient-style QA scenarios. These were used to evaluate the adaptability and conversational ability of PCaPLMM_SFT in natural patient queries (Table 7). Table 7 illustrates the model responses across these scenarios and not the best-case outputs. The full evaluation set of 2500 patient-style queries and representative failure cases and error-category analyses are provided in Table 5 and Multimedia Appendix 8.

**Table 7.** Example outputs from PCaPLMM_SFT^a^ across 5 lifestyle scenarios^b^.

Scenario	Patient query	Model response (excerpt)
Diet	How can I adjust my diet to better manage weight and maintain muscle strength while undergoing androgen deprivation therapy (ADT)?	During ADT, eat whole grains, fruits, vegetables, lean proteins (chicken, fish, beans, legumes), and healthy fats (avocados, nuts, seeds, olive oil); increase calcium and vitamin D from dairy, leafy greens, fish or supplements; drink plenty of water; limit processed foods, sugary drinks, and high-fat snacks; control portions with smaller plates; and combine diet with resistance training. Always consult with your healthcare provider before making significant changes to your diet or exercise regimen.
Exercise	What types of exercise are recommended for prostate cancer patients at different stages?	Recommended exercises include aerobic training ≥3 times/week, resistance training twice weekly, balance/core exercises, and impact-loading workouts such as jogging. Intensity and frequency should be tailored to treatment phase and fitness.
Psychological support	I’ve been feeling very anxious and stressed since being diagnosed with prostate cancer. What kind of psychological support can help me cope with this?	Support groups, individual counseling, Cognitive Behavioral Therapy, mindfulness, and online resources may help. Each patient is unique, so finding the right combination of emotional, social, and psychological support is essential for mental well-being.
Medication adherence	If I miss a dose or don’t take my prostate cancer meds on time, is that a big deal? What should I be worried about?	Non-adherence can reduce treatment efficacy, increase recurrence risk, and worsen outcomes. If facing challenges with your medication, talk to your provider to find solutions, such as adjusting schedules or using reminders.
Weight management	As a prostate cancer patient, I’m concerned that my current weight might increase my risk of heart problems. What are some safe ways for me to manage my BMI?	Combine healthy eating, exercise, sleep hygiene, and stress management. Consult healthcare professionals to develop a personalized plan for safe weight management and cardiovascular protection.

a PCaPLMM_SFT: Prostate Cancer Patient Lifestyle Management Model via Supervised Fine-Tuning.

b Responses shown in Table 7 are excerpts from automatically generated outputs. Full responses of the example outputs from PCaPLMM_SFT across 5 lifestyle scenarios are available in Multimedia Appendix 10. All answers underwent human review for semantic coherence, medical accuracy, and inclusion of safety disclaimers.

In the dietary management scenario, the query “How can I adjust my diet to better manage weight and maintain muscle strength while undergoing androgen deprivation therapy (ADT)?” elicited model-generated outputs recommending the consumption of whole grains, fruits, vegetables, lean proteins, and healthy fats, together with calcium and vitamin D supplementation. The model further advised limiting processed foods, sugary beverages, and high-fat snacks, combining dietary changes with resistance training, and underscored the need for individualized planning under physician supervision.

In exercise-related tasks, the model generated recommendations tailored to treatment phase and physical fitness level. For psychological support, the generated responses conveyed empathy and humanistic care. For medication adherence and weight management, the answers incorporated behavioral interventions, reminders, and risk prevention strategies. Overall, the model frequently generated safety-related statements such as “consult your physician” and “tailor interventions to individual assessment.” These statements reflected medical communication patterns learned during SFT and cautious response generation guided by the system prompt, rather than deterministic rule-based safety guardrails. PCaPLMM_SFT consistently outperformed Baichuan2-7B-Chat and demonstrated comparable or superior performance to GPT-3.5-Turbo across all 5 lifestyle scenarios in 2 independent evaluation rounds conducted by Qwen3-Max and DeepSeek-R1 (Table 8).

**Table 8.** Performance evaluation of 3 models across 5 lifestyle scenarios by referee LLMs^a^.

Scenario (round)	PCaPLMM_SFT^b^, mean (SD), 95% CI	Baichuan2-7B-Chat, mean (SD), 95% CI	GPT-3.5-Turbo, mean (SD), 95% CI	*P*_1_ value (FDR)^c^	|r_1_|	*P*_2_ value (FDR)	|*r*_2_|
Qwen3-Max							
Diet ①	18.979 (2.209), 18.785-19.173	16.743 (3.833), 16.407-17.080	18.916 (1.528), 18.782-19.050	<.001	0.484	.27	0.053
②	19.007 (2.186), 18.815-19.199	16.822 (3.548), 16.510-17.134	18.856 (1.496), 18.724-18.987	<.001	0.479	.86	0.008
Exercise ①	20.063 (2.321), 19.859-20.267	15.968 (3.650), 15.647-16.288	18.653 (1.552), 18.517-18.790	<.001	0.694	<.001	0.485
②	20.098 (2.295), 19.896-20.299	16.013 (3.618), 15.695-16.331	18.568 (1.583), 18.429-18.707	<.001	0.704	<.001	0.578
PS^d^ ①	19.147 (2.457), 18.931-19.363	15.445 (3.853), 15.106-15.783	18.813 (1.697), 18.664-18.962	<.001	0.627	.001	0.16
②	19.222 (2.396), 19.011-19.432	15.378 (4.067), 15.020-15.735	18.789 (1.733), 18.637-18.942	<.001	0.64	.02	0.104
MA^e^ ①	19.280 (1.720), 19.129-19.431	15.759 (2.647), 15.527-15.992	19.072 (1.287), 18.959-19.185	<.001	0.746	.001	0.157
②	19.246 (1.669), 19.100-19.393	15.857 (2.481), 15.639-16.075	18.959 (1.278), 18.846-19.071	<.001	0.745	.008	0.125
WM^f^ ①	19.379 (2.319), 19.175-19.583	16.099 (3.144), 15.822-16.375	18.443 (1.740), 18.290-18.596	<.001	0.659	<.001	0.338
②	19.503 (2.066), 19.321-19.684	15.944 (3.462), 15.640-16.248	18.566 (1.761), 18.412-18.721	<.001	0.641	<.001	0.312
DeepSeek-R1							
Diet ①	21.368 (1.836), 21.207-21.529	18.979 (3.901), 18.636-19.321	20.079 (1.674), 19.932-20.226	<.001	0.528	<.001	0.451
②	20.438 (2.258), 20.239-20.636	18.665 (3.766), 18.335-18.995	20.023 (1.597), 19.883-20.163	<.001	0.645	.02	0.116
Exercise ①	21.359 (2.376), 21.151-21.568	18.497 (4.511), 18.101-18.893	19.565 (2.529), 19.343-19.788	<.001	0.528	<.001	0.462
②	20.327 (2.776), 20.083-20.571	18.219 (4.166), 17.854-18.584	19.479 (2.488), 19.261-19.698	<.001	0.701	<.001	0.245
PS ①	21.515 (1.875), 21.351-21.680	19.483 (3.058), 19.214-19.751	19.990 (2.248), 19.793-20.188	<.001	0.515	<.001	0.45
②	20.942 (2.345), 20.736-21.148	18.888 (3.362), 18.593-19.183	19.905 (2.041), 19.725-20.084	<.001	0.76	<.001	0.339
MA ①	21.276 (2.137), 21.089-21.464	19.112 (3.211), 18.830-19.394	20.188 (2.099), 20.003-20.372	<.001	0.527	<.001	0.35
②	20.521 (1.665), 20.374-20.667	18.770 (3.676), 18.447-19.092	20.082 (2.112), 19.896-20.267	<.001	0.841	<.001	0.187
WM ①	21.196 (2.453), 20.980-21.411	18.654 (3.874), 18.313-18.994	19.445 (2.719), 19.206-19.684	<.001	0.495	<.001	0.431
②	20.540 (2.427), 20.327-20.753	18.319 (3.795), 17.986-18.651	19.369 (2.481), 19.151-19.587	<.001	0.736	<.001	0.338

a *P*_1_ and |*r*_1_| represent the comparative results between PCaPLMM_SFT and Baichuan2-7B-Chat, whereas *P*_2_ and |*r*_2_| correspond to the comparison between PCaPLMM_SFT and GPT-3.5-Turbo. *P* values are derived from 2-sided Mann–Whitney *U* tests and adjusted using the Benjamini–Hochberg procedure to control the false discovery rate. |*r*| denotes the absolute effect size, calculated from the standardized *Z* statistic and accompanied by 95% confidence intervals estimated via Fisher *Z* transformation.

b PCaPLMM_SFT: Prostate Cancer Patient Lifestyle Management Model via Supervised Fine-tuning.

c FDR: false discovery rate.

d PS: psychological support.

e MA: medication adherence.

f WM: weight management.

Under Qwen3-Max evaluation, PCaPLMM_SFT achieved significantly higher scores than Baichuan2-7B-Chat across all scenarios (diet, exercise, psychological support, medication adherence, and weight management; *P*<.001), with medium-to-large effect sizes (|*r*| ranging from 0.479 to 0.746). Compared with GPT-3.5-Turbo, PCaPLMM_SFT showed clear advantages in specific scenarios, particularly exercise, psychological support, and weight management (|*r*| ranging from 0.104 to 0.578), while maintaining comparable performance in others (eg, diet).

Under DeepSeek-R1 evaluation, the superiority of PCaPLMM_SFT over Baichuan2-7B-Chat was consistent and significant across all 5 scenarios, with the largest effect sizes observed in psychological support and medication adherence (*P*<.001; |*r*| ranging from 0.515 to 0.841). Comparisons against GPT-3.5-Turbo showed a similar favorable pattern, with PCaPLMM_SFT significantly outperforming the benchmark in most tasks (*P*<.001; |*r*| ranging from 0.116 to 0.462).

## Discussion

### Principal Findings

In this study, we developed and implemented PCaPLMM_SFT, a SFT LLM designed to support healthy lifestyle management for patients with prostate cancer. By integrating a structured literature-based KB, semantic retrieval, and instruction tuning, we generated high-quality QA pairs covering multiple lifestyle-related dialogue scenarios. Furthermore, 2 referee models were introduced for multidimensional evaluation. Results indicated that our model significantly outperformed the general-purpose models in key dimensions such as evidence alignment and comprehensibility, highlighting its potential for patient-clinician communication and personalized patient education. In addition to the automated referee evaluation, a small-scale human expert assessment was conducted to further validate the reliability of the referee model framework. Across both referee LLMs and 3 human experts, PCaPLMM_SFT was consistently ranked as the top-performing model in all lifestyle domains. Although the level of agreement varied across evaluators, the overall ranking trend remained stable, supporting the robustness of the comparative results.

For the base architecture, we selected Baichuan2-7B-Chat, which offers a balance between parameter scale and open accessibility, enabling targeted optimization and potential local deployment [14]. Unlike prior models that relied on authentic physician-patient dialogues for fine-tuning (eg, HuatuoGPT [26]) or expert-curated annotations (eg, Med-PaLM [23]), this study proposes an automated, literature-driven framework for training data generation. Through semantic chunking, structured indexing, and prompt-based reformulation, we constructed a large-scale, low-cost, and high-quality training corpus covering 5 major lifestyle scenarios. This approach not only enhances the controllability of medical knowledge but also addresses the long-standing challenges of data scarcity due to privacy restrictions and limited willingness for data sharing.

At the task level, PCaPLMM_SFT demonstrates an improved capacity for understanding and generating patient-style language. Compared with the document-focused generative models such as RadOnc-GPT [27], PCaPLMM_SFT emphasizes flexibility and empathy in open-ended dialogue, enabling multiturn interactions that incorporated emotional recognition, health guidance, and behavioral recommendations. These features improve communication effectiveness and user engagement in chronic disease management.

Traditional evaluation of medical QA systems has relied heavily on expert ratings, which are costly, subjective, and difficult to scale. With the emergence of the “LLM-as-a-Judge” paradigm [28-30], recent studies have demonstrated that LLM-based referees can provide stable, multidimensional evaluations at a fraction of the cost and time. Consistent with prior work—including validation by Zheng et al [31] in MT-Bench and Chatbot Arena, which showed that structured prompts and rubric-based scoring enabled LLM referees to approximate expert-level consistency—our results further support the feasibility of automated evaluation frameworks. The 2 referee models (Qwen3-Max and DeepSeek-R1) showed moderate to good agreement across rounds, indicating that standardized rubrics and controlled prompting can reduce evaluation variance. However, the lower stability of DeepSeek-R1 compared with Qwen3-Max may reflect model-specific differences in prompt sensitivity, reasoning behavior, and evidence integration; as a reasoning-oriented model, DeepSeek-R1 may be more affected by prompt wording, contextual structure, and the ordering or redundancy of retrieved evidence fragments, whereas Qwen3-Max may have followed the scoring rubric and formatted output more consistently [17,32,33]. These findings extend previous evidence and reinforce the role of automated referees as a practical, scalable tool for benchmarking domain-specific LLMs and guiding iterative model improvement.

In our expert-LLM consistency analysis, ICC values were in the moderate range, indicating that although the overall ranking trends across models were aligned, finer-grained scoring differed. This pattern is consistent with recent literature suggesting that human experts and LLM judges operate with distinct evaluative priors [10,31,34]. Human experts typically emphasize clinical risk, actionability, contextual relevance, and patient-specific feasibility [35]—dimensions inherently subjective and embedded in real-world clinical reasoning. In contrast, LLM referees tend to weight structured coherence, completeness, and evidence alignment more heavily.

Therefore, the observed discrepancy does not reflect disagreement in model performance but rather divergence in evaluation emphasis. These differences underscore that human and automated assessments are complementary rather than interchangeable. In this study, LLM referees were positioned as scalable, standardized, and reproducible tools for rubric-based evaluation rather than as substitutes for human clinical experts. They can efficiently assess evidence alignment, comprehensibility, relevance, empathy, and feasibility under unified criteria [36], but their scoring may still be influenced by prompt design, model-specific preferences, training corpora, and task framing. In contrast, human experts provide experience-based judgment on patient safety, individualized applicability [37,38], risk boundaries, and practical feasibility [39,40]. Integrating both perspectives enables a more comprehensive evaluation framework and mitigates the limitations associated with relying on either method.

### Strengths and Limitations

This study offers several methodological innovations beyond conventional SFT- or RAG-based biomedical LLM development: (1) Automated literature-to-QA generation pipeline: we proposed a fully automated workflow that transforms high-quality evidence into structured QA pairs, enabling efficient, standardized, and scalable construction of domain-specific training corpora. (2) A generalizable methodological paradigm for chronic disease management: the modular framework—integrating knowledge slicing, structured QA generation, and domain-adaptive SFT—can be readily extended to other chronic disease contexts, providing a reusable blueprint for developing patient education LLMs. (3) Evidence-grounded evaluation framework using a RAG-augmented referee LLM: we established a structured referee LLM–based multidimensional assessment system, enabling standardized evaluation of evidence alignment, safety, clarity, and relevance.

This study has several limitations. First, although the model’s responses integrate evidence-based medical knowledge, their effectiveness and safety have not been validated in real patient interactions; this remains a major limitation for a patient-facing system and an important prerequisite for clinical translation. In addition, the manual QA audit underscores the need for upstream quality control in automated literature-to-QA generation. Although revised and filtered samples were used for SFT, residual corpus noise may remain. Despite the additional interrater agreement analysis, full-corpus manual validation was not performed. Therefore, PCaPLMM_SFT should currently be regarded as a proof-of-concept and a patient education support tool rather than a stand-alone clinically deployable system. Second, despite using multiple referee models and incorporating human expert assessments, bias related to model architecture, training corpora, or corpus-generation procedures may still persist. Although the referee LLM outputs were examined through interround, intermodel, and human-LLM consistency analyses, the referee scores should be interpreted as auxiliary evaluation results rather than definitive expert adjudications. In particular, alternative QA generators were not systematically tested; therefore, potential generator-induced bias from Qwen3-Turbo may remain. Future studies should compare multiple QA generators and incorporate cross-model review or expert-annotated reference sets to further strengthen corpus diversity, evaluation robustness, and generalizability. Third, the human expert validation was based on a limited sample of 50 QA pairs across 5 lifestyle scenarios. Therefore, the expert evaluation should be interpreted as a supplementary consistency check rather than a fully representative assessment of the entire QA corpus or broader patient-facing use scenarios. Finally, as this work primarily focused on methodological development, real-world behavioral outcomes and long-term follow-up effects could not yet be assessed.

Future work should expand real-world interaction datasets and incorporate patient feedback to enhance external validity. Methodologically, applying stronger regularization, early stopping, and multisource corpora may further improve model generalization. In addition, the failure cases in Table 5 indicate that hallucination risks, including fabricated or irrelevant citations, cannot be fully eliminated by RAG and manual review alone; future work could integrate automated fact-checking modules for claim decomposition, citation verification, and evidence support assessment, while retaining expert review in clinically sensitive scenarios. Given the variability observed across referee LLMs and human experts, establishing a multilayer evaluation system that integrates LLM judges, clinicians, and patient users will be essential for improving the stability and safety assessment of model behavior. Finally, extending the proposed literature-to-QA pipeline and structured evaluation paradigm to other diseases represents an important direction for developing scalable, evidence-grounded patient education LLMs. Further validation with broader Chinese-language clinical and patient education resources is also warranted. Moreover, real-world RAG deployment involving multiple retrieved fragments and multiturn dialogue history may require adaptive chunk selection, history summarization, or longer-context models beyond the context window settings used in this study.

### Conclusions

As an SFT LLM developed for healthy lifestyle management in patients with prostate cancer, PCaPLMM_SFT supports the feasibility of combining domain-specific knowledge injection with structured QA-based training strategies. The model generated evidence-supported, comprehensible, and empathetic responses across lifestyle-related dialogue scenarios, suggesting its potential as a research prototype and patient education support tool under appropriate clinical oversight. This work also establishes a feasible and reproducible framework for developing disease-specific generative dialogue systems by integrating automated literature-to-QA construction and dual-layer evaluation. The methodology may provide guidance for adapting similar models to other chronic disease contexts. Moving forward, real-world patient interaction studies, iterative dataset refinement, and multistakeholder human-AI coevaluation will be needed before such systems can be considered for broader patient-facing deployment.

## Supplementary material

10.2196/92663 Multimedia Appendix 1 PubMed search strategy and inclusion and exclusion.

10.2196/92663 Multimedia Appendix 2 Detailed manual quality control evaluation results for sampled question-answer pairs.

10.2196/92663 Multimedia Appendix 3 Prompt templates for the large language model referees.

10.2196/92663 Multimedia Appendix 4 Five-dimension scoring framework.

10.2196/92663 Multimedia Appendix 5 Expert evaluation form for multiple model outputs on lifestyle management in patients with prostate cancer.

10.2196/92663 Multimedia Appendix 6 Classification of manual quality control outcomes for the sampled question-answer pairs.

10.2196/92663 Multimedia Appendix 7 Representative failure cases during manual quality control.

10.2196/92663 Multimedia Appendix 8 Proportion of different error types across models.

10.2196/92663 Multimedia Appendix 9 Human expert evaluation scores for candidate models.

10.2196/92663 Multimedia Appendix 10 Full responses of the example question-answer pairs generated by PCaPLMM_SFT (Prostate Cancer Patient Lifestyle Management Model via Supervised Fine-Tuning).

## Abbreviations

ADT: androgen deprivation therapy
FAISS: Facebook Artificial Intelligence Similarity Search
ICC: intraclass correlation coefficient
KB: knowledge base
LLM: large language model
PCaPLMM_SFT: Prostate Cancer Patient Lifestyle Management Model via Supervised Fine-tuning
PRISMA: Preferred Reporting Items for Systematic Reviews and Meta-Analyses
QA: question-answer
RAG: retrieval-augmented generation
SFT: supervised fine-tuning
