# Supplementary material for: A Supervised Fine-Tuned Large Language Model for Lifestyle Management in Patients With Prostate Cancer: Development and Evaluation Study
Source: J Med Internet Res. 2026 Jul 21;28:e92663. doi: 10.2196/92663 (PMC13387489; doi:10.2196/92663)
Supplement: Multimedia Appendix 1 [file jmir-v28-e92663-s001.docx]

**Multimedia Appendix 1. PubMed search strategy and inclusion/exclusion criteria**

**Search Strategy**

| **PubMed Search Strategy** |
| --- |
| ((((((((((((((((Prostatic Neoplasms[Title/Abstract]) OR (Prostate Neoplasms[Title/Abstract])) OR (Neoplasms, Prostate[Title/Abstract])) OR (Neoplasm, Prostate[Title/Abstract])) OR (Prostate Neoplasm[Title/Abstract])) OR (Neoplasms, Prostatic[Title/Abstract])) OR (Neoplasm, Prostatic[Title/Abstract])) OR (Prostatic Neoplasm[Title/Abstract])) OR (Prostate Cancer[Title/Abstract])) OR (Cancer, Prostate[Title/Abstract])) OR (Cancers, Prostate[Title/Abstract])) OR (Prostate Cancer[Title/Abstract])) OR (Cancer of the Prostate[Title/Abstract])) OR (Prostatic Cancer[Title/Abstract])) OR (Cancer, Prostatic[Title/Abstract])) OR (Cancers, Prostatic[Title/Abstract])) OR (Prostatic Cancers[Title/Abstract]) |
| AND |
| ((lifestyle[Title/Abstract]) OR (life styles[Title/Abstract]) OR (life style[Title/Abstract]) OR (risk factor[Title/Abstract]) OR (nutri*[Title/Abstract]) OR (Physical activity[Title/Abstract]) OR (vitamin[Title/Abstract]) OR (smok*[Title/Abstract]) OR (wine[Title/Abstract]) OR (tea[Title/Abstract]) OR (coffee[Title/Abstract]) OR (diet*[Title/Abstract]) OR (dairy[Title/Abstract]) OR (nutrient*[Title/Abstract]) OR (alcohol[Title/Abstract]) OR (fruit[Title/Abstract]) OR (vegetable[Title/Abstract]) OR (environment*[Title/Abstract]) OR (sleep[Title/Abstract]) OR (social[Title/Abstract]) OR (sun exposure[Title/Abstract]) OR (folate[Title/Abstract]) OR (birth weight[Title/Abstract]) OR (carotene[Title/Abstract]) OR (lifestyl*[Title/Abstract]) OR (fiber[Title/Abstract]) OR (fried[Title/Abstract]) OR carbohydrate[Title/Abstract] OR (sedenta*[Title/Abstract]) OR (depress*[Title/Abstract]) OR (stress*[Title/Abstract]) OR (behavi*[Title/Abstract])) |

**Eligibility and Exclusion Criteria**

Studies were eligible for inclusion if they met the following criteria: (1) **Population**: participants were patients with prostate cancer or individuals at high risk of prostate cancer; (2) **Intervention/Exposure**: the study assessed at least one lifestyle-related factor, including but not limited to diet, nutrition, physical activity, exercise, smoking, alcohol consumption, obesity, sleep, circadian rhythm, sedentary behavior, stress, or other modifiable lifestyle behaviors; (3) **Comparison**: studies included a relevant comparison group or allowed comparison across different exposure levels, intervention groups, or time points; (4) **Outcomes**: studies reported prostate cancer-related outcomes, including incidence, disease progression, recurrence, survival, mortality, treatment-related outcomes, symptoms, or quality of life; and (5) **Study design**: cohort studies, case-control studies, cross-sectional studies, clinical trials, systematic reviews, and meta-analyses published in English between 2015 and 2025 were considered.

Studies were excluded if they were non-human studies, did not focus on prostate cancer as a primary research subject, did not assess lifestyle-related factors, focused exclusively on pharmacological treatment, surgery, radiotherapy, chemotherapy, or other clinical treatments without lifestyle-related components, or were single case reports. For multiple publications based on the same study population with overlapping exposures and outcomes, only the most complete, most recent, or longest follow-up report was retained.
