# Supplementary material for: A Supervised Fine-Tuned Large Language Model for Lifestyle Management in Patients With Prostate Cancer: Development and Evaluation Study
Source: J Med Internet Res. 2026 Jul 21;28:e92663. doi: 10.2196/92663 (PMC13387489; doi:10.2196/92663)
Supplement: Multimedia Appendix 2 [file jmir-v28-e92663-s002.docx]

**Multimedia Appendix 2. Detailed manual quality-control evaluation results for sampled QA pairs**

The 2,400 manually reviewed QA pairs represented 5.62% of the pre-final QA candidate corpus of 42,705 QA pairs used for quality-control auditing, prior to correction, filtering, and final dataset inclusion. Under simple-random-sampling assumptions, a conservative normal approximation for a sample proportion, with p = 0.50 and finite-population correction, yields a nominal approximate 95% maximum margin of error of ±1.94 percentage points. This audit sample supports corpus-level estimation of frequent quality-control problems and relatively common error categories. However, because rare events and clinically heterogeneous edge cases may remain underrepresented, the audit should not be interpreted as exhaustive validation of rare safety failures, low-frequency lifestyle subtopics, or complex patient subgroups.

Table S1. Reliability coefficients for 1–5 dimensional quality-control scores.

| Dimension | ICC(3,k) | ICC(A,k) | Pearson r | Spearman rho |
| --- | --- | --- | --- | --- |
| Evidence correctness | 0.738 | 0.623 | 0.597 | 0.482 |
| Content completeness | 0.735 | 0.636 | 0.589 | 0.509 |
| Personalization | 0.671 | 0.655 | 0.508 | 0.450 |
| Clarity and communication | 0.812 | 0.745 | 0.689 | 0.622 |
| Practicality and actionability | 0.790 | 0.696 | 0.653 | 0.624 |
| Overall dimension-level scores | 0.780 | 0.712 | 0.640 | 0.600 |
| *Note:* ICC(3,k) estimates consistency for the average of the two fixed evaluation versions; ICC(A,k) estimates absolute agreement. The overall row pools all paired dimension-level scores. | | | | |

In paired analysis of the two post-evaluation review files, overall dimensional score reliability was moderate-to-good (ICC[3,k]=0.780; ICC[A,k]=0.712), while categorical disposition agreement was lower (unweighted kappa=0.161; quadratic weighted kappa=0.378), reflecting stricter revision thresholds in one evaluation version.

Table S2. Inter-rater agreement metrics for the manual QA quality-control audit.

| Comparison | Metric type | Estimate | 95% CI |
| --- | --- | --- | --- |
| Reviewer 1 vs Reviewer 2 | ICC(3, k) | 0.780 | [0.772, 0.789] |
|  | ICC(A, k) | 0.712 | [0.701, 0.724] |
|  | Pearson r | 0.640 | - |
|  | Spearman ρ | 0.600 | - |
|  | Exact same 1-5 score | 40.8% | - |
|  | Difference <=1 point | 87.4% | - |
