# Supplementary material for: A Supervised Fine-Tuned Large Language Model for Lifestyle Management in Patients With Prostate Cancer: Development and Evaluation Study
Source: J Med Internet Res. 2026 Jul 21;28:e92663. doi: 10.2196/92663 (PMC13387489; doi:10.2196/92663)
Supplement: Multimedia Appendix 4 [file jmir-v28-e92663-s004.docx]

**Multimedia Appendix 4. Five-dimension scoring framework**

| **Dimensions** | **Description** | **Scoring Notes** |
| --- | --- | --- |
| Evidence alignment | Whether the response is accurate, scientifically justified, and aligned with current evidence from high-quality clinical research. | 5 points: Fully aligned with high-quality empirical evidence, with precise terminology and scientifically coherent reasoning.  4 points: Generally consistent with established clinical evidence, with only minor limitations in evidentiary depth.  3 points: Partially supported by current evidence, though containing minor inaccuracies or overly generalized statements.  2 points: Weakly grounded in empirical research, with vague phrasing or unclear evidentiary rationale.  1 point: Contradicts established clinical evidence or contains misleading and potentially unsafe medical statements. |
| Comprehensibility | Whether the response is clearly structured, easy to understand, and adequately covers the scenario addressed by the question. | 5 points: Clearly structured, fluent in language, and comprehensively addresses the question context.  4 points: Well organized and generally covers the key points.  3 points: Average clarity, with partial omissions or areas of less fluent expression.  2 points: Poor logic with incomplete coverage.  1 point: Disorganized content with unclear expression, making it difficult to understand. |
| Relevance | Whether the response fully considers the patient’s individual characteristics and cultural background. | 5 points: Highly personalized, fully integrating the patient’s characteristics and contextual factors.  4 points: Shows a certain degree of specificity and reflects the background reasonably well.  3 points: Partially reflects individual differences.  2 points: Generalized content with poor adaptability.  1 point: The content is highly inconsistent with the patient’s background. |
| Empathy | Whether the response demonstrates respect, care, and empathy. | 5 points: Warm and natural tone with clear empathy.  4 points: Friendly tone with some emotional expression.  3 points: Neutral tone, lacking warmth.  2 points: Stiff or indifferent tone.  1 point: Mechanical tone with no emotion or signs of disrespect. |
| Feasibility | Whether the recommendations are specific and clear, and appropriately alert the patient to potential risks. | 5 points: Recommendations are specific and clear, with comprehensive risk reminders.  4 points: Recommendations are useful and the risk reminders are reasonably addressed.  3 points: Recommendations are moderately actionable, but the risk reminders are incomplete.  2 points: Recommendations are vague or impractical, with insufficient risk reminders.  1 point: No substantive recommendations and no mention of potential risks. |
