# Supplementary material for: A Supervised Fine-Tuned Large Language Model for Lifestyle Management in Patients With Prostate Cancer: Development and Evaluation Study
Source: J Med Internet Res. 2026 Jul 21;28:e92663. doi: 10.2196/92663 (PMC13387489; doi:10.2196/92663)
Supplement: Multimedia Appendix 5 [file jmir-v28-e92663-s005.docx]

**Multimedia Appendix 5. Expert evaluation form for multiple model outputs on lifestyle management in prostate cancer patients (example QA)**

These candidate responses are examples for evaluation and should not be interpreted as clinical recommendations.

| **Question 1** | **I was recently diagnosed with prostate cancer, and my doctor recommended increasing my intake of plant-based foods. Since I usually prefer meat, I would like to know how to achieve a balanced diet.** | **Evidence alignment** | **Comprehensibility** | **Relevance** | **Empathy** | **Feasibility** |
| --- | --- | --- | --- | --- | --- | --- |
| Response 1 | Hello! When it comes to maintaining a balanced diet, you may consider the following points:  Diversify your intake: Try to include vegetables, fruits, whole grains, and lean protein on your plate. This helps you obtain a variety of nutrients and avoid relying too much on a single type of food.  Moderately increase plant-based foods: Aim to eat at least five servings of different non-starchy vegetables and fruits each day. This helps improve fiber intake and supports digestive health.  Choose healthy protein sources: Prefer fish, skinless poultry, and plant-based proteins such as legumes, and reduce your consumption of red and processed meats.  Control portion sizes: Pay attention to the amount of food you eat at each meal to avoid overeating. Reduce cooking time and avoid frying to lower calorie density.  Stay hydrated: Drink plenty of water and reduce intake of sugary beverages.  By following these steps, you can gradually adjust your eating habits to better meet your body's needs. If you have any questions or need further help, please feel free to consult a professional doctor or dietitian. Wishing you good health! |  |  |  |  |  |
| Response 2 | Yes, excessive meat consumption may affect prostate health. It is recommended to moderately reduce the intake of red and processed meats, and increase the proportion of plant-based foods such as vegetables, fruits, whole grains, and nuts to maintain a balanced diet. In addition, eating foods rich in zinc, vitamin E, and selenium—such as seafood, nuts, and fruits and vegetables—can also help maintain prostate health. It is best to engage in regular physical activity and maintain healthy lifestyle habits to help prevent prostate problems. If you have prostate-related health concerns, it is advisable to consult a doctor or dietitian. |  |  |  |  |  |
| Response 3 | I suggest you try the following approaches to balance your diet:  Gradually reduce meat consumption: For example, reduce one meat-based dinner per week. This can help you gradually adapt to plant-based foods and avoid discomfort caused by abrupt dietary changes.  Increase sources of plant-based proteins: Try eating more legumes, nuts, seeds, and whole grains to meet your nutritional needs.  Explore new cooking methods: Use different methods like steaming, baking, and stewing to reduce fat intake while preserving nutritional value.  Combine diverse ingredients: Include a variety of plant-based foods in each meal to ensure intake of different nutrients.  Learn the benefits of plant-based foods: Understand the nutritional value of plant-based foods—such as being rich in fiber, antioxidants, and healthy fats—which will encourage you to make more positive choices.  Be patient and persistent: Changing eating habits takes time and effort. Don’t expect immediate results. Stay patient and keep adjusting your diet gradually. |  |  |  |  |  |
| **Question 2** | **As a prostate cancer patient, what high-risk exercises should I avoid?** | **Evidence alignment** | **Comprehensibility** | **Relevance** | **Empathy** | **Feasibility** |
| Response 1 | Hello! As a prostate cancer patient, you should pay attention to the following points regarding exercise:  Avoid high-intensity exercise: Since prostate cancer patients may experience fatigue or other symptoms, high-intensity activities should be avoided. For example, high-intensity exercises like running or swimming may worsen fatigue.  Avoid impact sports: Activities like football or basketball may increase the risk of fractures.  Avoid strenuous exercise: Such as sprinting or jumping, as they may increase the load on the heart.  Avoid standing or walking for long periods: This may worsen leg fatigue.  Avoid vigorous exercise: Vigorous activity may increase muscle fatigue and pain.  Avoid overexertion: Any activity that exceeds the body's capacity may cause excessive fatigue, potentially delaying recovery.  It is recommended to choose low-intensity, low-impact exercises, such as walking, yoga, or tai chi. These are not only suitable for prostate cancer patients but also help improve physical condition. Before starting any new exercise program, please be sure to consult your doctor or physical therapist to ensure safety and appropriateness. I hope these suggestions are helpful! |  |  |  |  |  |
| Response 2 | As a prostate cancer patient, you should avoid exercises that may increase your risk, including:  High-impact exercises: Running, jumping, football, etc., may increase the risk of fractures.  Exercises requiring deep squats or strain: Such as weightlifting or certain yoga poses, which may increase the risk of prostate congestion.  Exercises that involve long periods in the same position: Yoga, tai chi, etc., may increase pain or discomfort if not performed with adjustments.  Exercises involving intense effort: Such as running or cycling, which may put extra burden on the heart and lungs.  Exercises that may cause strong emotional responses: Boxing, wrestling, etc., which may raise psychological stress.  However, this does not mean you cannot exercise at all. In fact, appropriate exercise can improve your quality of life, reduce pain, increase strength, and enhance immune system function. You may choose low-impact activities such as walking, swimming, or cycling, which are relatively safe for prostate cancer patients. Always consult your doctor before starting any new exercise plan. |  |  |  |  |  |
| Response 3 | As a prostate cancer patient, you should avoid the following high-risk activities:  High-impact exercises: Avoid intense impact sports such as running, jumping, basketball, etc., to prevent excess pressure and stimulation on the prostate.  Cycling: Prolonged cycling may increase pressure and friction on the prostate area. Avoid long rides or choose cycling methods that reduce pressure, such as using cushioned or shock-absorbing seats.  Weightlifting: Avoid heavy weightlifting to prevent increased abdominal pressure and muscle tension that may negatively affect the prostate.  Prolonged urine retention: Avoid holding urine for long periods; timely urination reduces pressure and stimulation to the prostate and helps protect its health.  Please note: If you experience any discomfort or symptoms such as frequent urination, urgency, pain, or hematuria during or after exercise, you should seek medical attention promptly and consult your doctor about suitable exercise types. Maintaining moderate physical activity and healthy lifestyle habits is essential for recovery and well-being in prostate cancer patients. |  |  |  |  |  |
| **Question 3** | **When facing the stress of prostate cancer, I feel very anxious. Are there any psychological support methods you could recommend for me?** | **Evidence alignment** | **Comprehensibility** | **Relevance** | **Empathy** | **Feasibility** |
| Response 1 | Stress affects physical health, including disease recovery. Persistent stress may affect the immune system and other bodily functions. Therefore, reducing stress is very important for disease recovery. Here are some psychological support measures that can help you reduce stress:  Seek professional psychological counseling: Have face-to-face or online counseling with a psychological expert to help you understand and handle stress.  Learn relaxation techniques: Try yoga, meditation, deep breathing, or warm baths to help relax your body and mind.  Maintain a healthy lifestyle: Exercise regularly, eat a balanced diet, get enough sleep, and avoid bad habits such as heavy drinking and smoking.  Communicate with family and friends: Share your feelings and concerns, and seek support and understanding.  Cultivate hobbies and interests: Participate in activities you enjoy to make yourself feel happy and satisfied.  I hope these suggestions are helpful to you, and I also recommend that you seek professional help in a timely manner and take care of your mental health. Wishing you relief from stress soon and good physical and mental health! |  |  |  |  |  |
| Response 2 | Hello! First of all, thank you for consulting me about this problem. Facing the pressure of cancer indeed makes people feel anxious and uneasy, but please believe that there are many psychological support methods that can help you relieve these emotions. Here are some suggestions:  Seek professional psychological counseling: Consider contacting a professional counselor or psychiatrist who can provide targeted psychological support and intervention. Cognitive Behavioral Therapy (CBT) is an effective method that can help you change negative thinking patterns and improve coping ability.  Join support groups: Communicating with people who are experiencing similar situations can bring resonance and support. You can look for local prostate cancer patient support groups or join relevant online communities.  Practice mindfulness meditation: Mindfulness meditation helps reduce stress and anxiety and improves psychological health. Spending a few minutes each day doing deep breathing or meditation may help you.  Maintain social connections: Keep good communication with family and friends and share your feelings and experiences. Social support is very important for relieving psychological stress.  Obtain information and support: Learn about prostate cancer, including treatment options and prognosis, which can reduce fear caused by uncertainty. You can also ask your doctor whether there are experience-sharing stories from other patients.  I hope these suggestions can help you. Please remember that paying attention to mental health is equally important, and seeking help in time is a very positive action. Wishing you recovery of both physical and mental health soon! |  |  |  |  |  |
| Response 3 | Of course there is. When facing the pressure of prostate cancer, seeking psychological support is very important. Here are some suggestions that can help you cope with stress and anxiety:  Talk to your doctor or therapist: Discuss your feelings and concerns with your doctor or professional therapist. They can provide professional advice and support to help you cope with stress.  Join support groups: Participate in support groups for prostate cancer patients and their families, and share experiences and feelings with others facing similar problems. This can help you understand others’ experiences and find resonance and support.  Learn to relax: Try learning relaxation techniques such as deep breathing, progressive muscle relaxation, or meditation. These methods can help you stay calm and focused when facing stress.  Maintain a positive mindset: Try to maintain a positive attitude and focus on the good things in life. You can try listing things you are grateful for to help you focus on the positive side.  Build a support network: Share your feelings with family, friends, and colleagues and seek their support and understanding. A strong support network can help you get through difficult times.  Maintain a healthy lifestyle: Maintaining a healthy lifestyle is crucial for coping with stress and anxiety. Ensure a balanced diet, appropriate exercise, and adequate sleep.  Seek professional help: If you find it difficult to cope with stress and anxiety, consider seeking psychological treatments such as Cognitive Behavioral Therapy (CBT) or Acceptance and Commitment Therapy (ACT). These therapies can help you change negative thinking patterns and improve stress-coping abilities.  Please remember that you are not alone, and there are many people and resources that can provide support for you. Be brave in seeking help and try your best to cope with the challenges brought by prostate cancer. |  |  |  |  |  |
| **Question 4** | **I need to take medication long-term to manage my prostate cancer. How can I improve my medication adherence?** | **Evidence alignment** | **Comprehensibility** | **Relevance** | **Empathy** | **Feasibility** |
| Response 1 | To improve long-term medication adherence, you can try the following methods:  Take medication on time: Establish a regular medication schedule, set reminder alarms or use a pill organizer to ensure timely medication.  Take medication according to medical advice: Strictly follow the doctor’s recommendations and prescriptions, and do not increase or decrease doses or change the way you take medications on your own.  Communicate with your doctor: Communicate regularly with your doctor to understand the effects, side effects, and precautions of the medications, and provide timely feedback about your medication use.  Seek support: You can seek help from family, friends, or support groups to jointly monitor your medication situation and increase motivation.  Establish healthy habits: Maintain good lifestyle habits such as regular sleep, balanced diet, and moderate exercise, which help improve the effectiveness of medications.  Psychological support: If you have emotional issues or anxiety, you may seek psychological counseling or support; maintaining a positive mindset is beneficial for long-term medication adherence.  I hope the above suggestions can help you improve your medication adherence and maintain good health. If you have any questions, please consult your doctor in time. I wish you good health! |  |  |  |  |  |
| Response 2 | Improving medication adherence is key to ensuring treatment effectiveness and preventing side effects. The following suggestions may help you improve medication adherence:  Understand your medications: Become familiar with the medications prescribed by your doctor, including their effects, side effects, contraindications, and interactions. This will help you better understand why you need to take them and how to use them safely.  Set clear goals: Discuss your treatment goals with your doctor and set clear, measurable, achievable, relevant, and time-limited (SMART) goals. This will help you stay motivated and focused.  Make a plan: Develop a daily plan to ensure you take your medication on time. This may include taking medications at a fixed time period, placing medications in a visible location, or using reminder tools (such as mobile apps or alarms).  Follow the doctor’s advice: Make sure you follow the doctor’s instructions and recommendations. If you have any questions about the treatment plan, contact your doctor or pharmacist at any time.  Maintain communication: Stay in regular contact with your doctor and pharmacist so they can understand your condition and treatment progress. This helps ensure your treatment plan is still effective and allows for timely adjustments.  Manage interfering factors: Identify factors that may affect your adherence, such as other illnesses, lifestyle stress, or other medications. Seek support to overcome these challenges, such as sharing your treatment plan with family and friends or seeking professional help if needed.  Maintain a positive mindset: Stay positive and believe that you can successfully achieve your treatment goals. Do not be discouraged when encountering difficulties; instead, seek help and support.  Celebrate success: When you reach a treatment goal or achieve an important medication-adherence milestone, remember to reward yourself. This will help keep you motivated.  Be patient: Improving your health may take time and effort. Be patient and believe you will gradually see improvement.  Keep learning and adjusting: Over time, your condition and treatment needs may change. Stay open-minded and adjust your treatment plan and adherence strategies as needed. |  |  |  |  |  |
| Response 3 | To improve your medication adherence, you can try the following methods:  Establish a regular medication habit: Take your medication at a fixed time each day, such as combining it with daily activities (like breakfast or lunch). This can help you remember medication times more easily.  Use reminder tools: Set phone alarms or use dedicated medication-management apps to remind you when it is time to take your medication. These tools can help you avoid missing any dose.  Understand your medications: Clearly understand the medications you are taking and their mechanisms of action. This can help you better understand why taking them on time is important.  Seek support: Share your treatment plan with family or friends so they can help supervise and remind you. In addition, join patient support groups to communicate with others experiencing similar situations.  Personalized information: Obtain detailed instructions and medication advice tailored to your personal situation. This information should include your diagnosis, treatment plan, and specific guidance on how to follow medical instructions.  Regular follow-ups: Maintain regular communication with your doctor and provide timely feedback about any problems encountered while taking medications. The doctor can adjust the dosage or treatment plan according to your specific situation.  By using the above methods, you can manage your medication treatment more effectively and improve medication adherence. If you still encounter difficulties, it is recommended to consult professional medical personnel for further help. |  |  |  |  |  |
| **Question 5** | **My weight has been increasing. Will this affect the effectiveness of my prostate cancer treatment? How should I manage my weight?** | **Evidence alignment** | **Comprehensibility** | **Relevance** | **Empathy** | **Feasibility** |
| Response 1 | Weight gain may affect the treatment effect of prostate cancer, because being overweight or obese increases the risk of cancer recurrence and complications. To manage weight, it is recommended that you take the following measures:  Dietary control: Control dietary intake, avoid high-calorie, high-fat, and high-sugar foods, and eat more vegetables, fruits, whole grains, lean meat, and low-fat dairy products.  Exercise: Perform at least 150 minutes of aerobic exercise per week, such as brisk walking, jogging, or swimming, which can help control weight and improve physical health.  Control alcohol consumption: Limit alcohol intake; excessive drinking is associated with weight gain and an increased risk of prostate cancer.  Regular weight monitoring: Regularly check weight changes, adjust diet and exercise plans in a timely manner, and maintain a healthy weight. If you have prostate cancer or other health problems, it is recommended to consult a doctor or professional nutritionist to develop a health management plan suitable for your personal situation. |  |  |  |  |  |
| Response 2 | Hello! Your concerns are very reasonable. Studies have shown that being overweight or obese may affect the treatment effect and prognosis of prostate cancer. For example, obesity may increase the risk of surgical complications and may affect the effect of radiotherapy. In addition, some studies have found that obesity may be associated with more aggressive prostate cancer and disease-specific mortality.  To help you better manage your weight, the following are some suggestions:  Healthy diet: Try to adopt a balanced diet plan, eat more vegetables, fruits, whole grains, and lean protein. Reduce the intake of high-calorie and high-fat foods, especially saturated fats and trans fats.  Regular exercise: Perform at least 150 minutes of moderate-intensity aerobic exercise per week (such as brisk walking or swimming) or 75 minutes of high-intensity aerobic exercise. At the same time, add strength training at least two days per week.  Portion control: Pay attention to the portion size of each meal and avoid overeating. You can use smaller tableware to help control food portions.  Stay hydrated: Drink enough water every day and avoid sugary drinks and high-calorie beverages.  Mental health: Maintain a positive attitude, and seek psychological counseling if necessary to cope with stress.  Regular monitoring: Regularly measure weight and waist circumference to observe trends. If conditions permit, you can participate in professional weight management programs.  Please be sure to discuss these plans with your doctor to ensure that they are suitable for your specific situation. Hope these suggestions are helpful to you! |  |  |  |  |  |
| Response 3 | Weight gain may affect the treatment effect of prostate cancer. Studies have shown that obesity may be related to an increased risk of prostate cancer and may also affect the effectiveness of some treatment drugs. Therefore, maintaining a healthy weight is very important for prostate cancer patients.  To manage weight, you can try the following methods:  Balanced diet: Ensure that your diet includes enough protein, fiber, vitamins, and minerals. Avoid excessive intake of high-calorie, high-fat, and high-sugar foods. At the same time, pay attention to the size of the plate to avoid overeating.  Regular exercise: Perform at least 150 minutes of moderate-intensity aerobic exercise per week, such as brisk walking, swimming, or cycling. In addition, you can also do strength training to enhance muscle and bone health.  Reduce sedentary time: Prolonged sitting may lead to weight gain and lack of exercise. Try to get up and move every hour, such as walking, stretching, or taking a water break.  Adequate sleep: Ensure 7–9 hours of high-quality sleep every night. Good sleep helps control weight and relieve stress.  Record diet and exercise: By recording daily diet and exercise, you can better understand your lifestyle habits and find areas that need improvement.  Seek professional advice: If you find it difficult to manage your weight, you can seek help from a nutritionist, doctor, or other professionals. They can provide personalized advice and support based on your specific situation.  Please note that these suggestions are not professional medical advice for individual situations. If your weight seriously affects prostate cancer treatment, please consult your doctor or professional medical personnel. |  |  |  |  |  |
