# Supplementary material for: A Supervised Fine-Tuned Large Language Model for Lifestyle Management in Patients With Prostate Cancer: Development and Evaluation Study
Source: J Med Internet Res. 2026 Jul 21;28:e92663. doi: 10.2196/92663 (PMC13387489; doi:10.2196/92663)
Supplement: Multimedia Appendix 6 [file jmir-v28-e92663-s006.docx]

**Multimedia Appendix 6.** **Classification of manual quality-control outcomes for the sampled QA pairs**

| **Decision Category** | **Number of Samples** | **Percentage (%)** | **Description** |
| --- | --- | --- | --- |
| Minor Edit | 1,217 | 50.71% | Minor revisions such as wording clarification or structural adjustment |
| Major Edit | 590 | 24.58% | Substantial revisions required due to evidence-discordant statements, factual inaccuracies, or inadequate logical justification |
| Accept | 547 | 22.79% | Medically accurate and well-structured responses requiring no edits |
| Reject | 46 | 1.92% | Irreparable issues such as misleading content, major medical errors, or off-topic |
| Total | 2,400 | 100.00% |  |
