# Supplementary material for: A Supervised Fine-Tuned Large Language Model for Lifestyle Management in Patients With Prostate Cancer: Development and Evaluation Study
Source: J Med Internet Res. 2026 Jul 21;28:e92663. doi: 10.2196/92663 (PMC13387489; doi:10.2196/92663)
Supplement: Multimedia Appendix 7 [file jmir-v28-e92663-s007.docx]

**Multimedia Appendix 7. Representative failure cases during manual quality control**

| **Error Type** | **Original Model**  **Output (Excerpt)** | **Problem Description** | **Corrected Example** |
| --- | --- | --- | --- |
| Factual inconsistencies with existing research evidence (e.g., outdated, unproven, or falsified claims). | “Vitamin E supplementation can reduce the risk of prostate cancer progression.” | The statement contradicts current evidence. Large randomized trials and meta-analyses have not shown any benefit of vitamin E supplementation for preventing or slowing prostate cancer progression. | “There is no high-quality clinical evidence demonstrating that vitamin E supplementation slows the progression of prostate cancer. Most large trials have found no preventive or therapeutic benefit, and patients are generally encouraged to obtain antioxidants from natural dietary sources rather than supplements..” |
| Overly general or vague lifestyle recommendations lacking actionable details | “Maintain a balanced diet and eat more healthy foods.” | The suggestion lacks specificity (no amounts, frequencies, or examples). Patients cannot operationalize the advice. | “Aim for at least 400 g of vegetables and fruits per day. Limit processed meat to no more than once per week. Replace part of your red meat intake with fish or legumes, and prioritize steaming or boiling instead of frying.” |
| Incomplete coverage of key lifestyle information (e.g., missing safety notes, doses, or stage-specific considerations) | “Engage in aerobic exercise to improve your health.” | Missing essential components: exercise frequency, intensity, duration, and safety considerations. Does not distinguish postoperative, radiotherapy, or ADT phases. | “During postoperative recovery (2–6 weeks), perform low- to moderate-intensity walking three times per week for 20–30 minutes (40–60% of your maximum heart rate). Gradually increase duration as tolerated. If you are experiencing radiotherapy-related fatigue, shorten sessions and add rest intervals.” |
| Imprecise, overly technical, or non–patient-centered language | “A diet high in antioxidant components helps modulate oxidative-stress-related signaling pathways.” | Excessively technical, not adapted to patient reading level; lacks empathic or supportive tone. | “Foods rich in natural antioxidants—such as blueberries, spinach, and tomatoes—may support overall health. A simple way to start is adding one serving of fruit or leafy greens to your daily meals.” |
