# Supplementary material for: A Supervised Fine-Tuned Large Language Model for Lifestyle Management in Patients With Prostate Cancer: Development and Evaluation Study
Source: J Med Internet Res. 2026 Jul 21;28:e92663. doi: 10.2196/92663 (PMC13387489; doi:10.2196/92663)
Supplement: Multimedia Appendix 8 [file jmir-v28-e92663-s008.docx]

**Multimedia Appendix 8**. **Proportion of different error types across models**

| **Error Type** | **PCaPLMM_SFT** | **Baichuan2-7B-chat** | **GPT-3.5-Turbo** |
| --- | --- | --- | --- |
| Evidence misalignment | 3.68% | 20.66% | 5.60% |
| Insufficiently actionable advice | 9.28% | 17.06% | 10.22% |
| Imprecise or non–patient-centered expression | 5.68% | 12.82% | 1.42% |
| Hallucination | 5.32% | 12.88% | 4.14% |
|  |  |  |  |
