# Supplementary material for: A Supervised Fine-Tuned Large Language Model for Lifestyle Management in Patients With Prostate Cancer: Development and Evaluation Study
Source: J Med Internet Res. 2026 Jul 21;28:e92663. doi: 10.2196/92663 (PMC13387489; doi:10.2196/92663)
Supplement: Multimedia Appendix 9 [file jmir-v28-e92663-s009.docx]

**Multimedia Appendix 9. Human expert evaluation scores for candidate models**

| **Dimension** | **PCaPLMM_SFT**  **(Mean ± SD [95% CI])** | **Baichuan2-7B-chat**  **(Mean ± SD [95% CI])** | **GPT-3.5-Turbo**  **(Mean ± SD [95% CI])** |
| --- | --- | --- | --- |
| **Expert 1** |  |  |  |
| Evidence alignment | 3.880 ± 0.627 [3.706, 4.054] | 3.540 ± 0.885 [3.295, 3.785] | 3.380 ± 0.725 [3.179, 3.581] |
| Comprehensibility | 4.660 ± 0.593 [4.496, 4.824] | 4.440 ± 1.072 [4.143, 4.737] | 4.320 ± 0.913 [4.067, 4.573] |
| Relevance | 4.500 ± 0.909 [4.248, 4.752] | 4.180 ± 1.351 [3.806, 4.554] | 4.120 ± 1.081 [3.820, 4.420] |
| Empathy | 4.080 ± 0.665 [3.896, 4.264] | 3.340 ± 0.798 [3.119, 3.561] | 3.420 ± 0.575 [3.261, 3.579] |
| Feasibility | 3.940 ± 0.740 [3.735, 4.145] | 3.580 ± 1.032 [3.294, 3.866] | 3.200 ± 1.088 [2.898, 3.502] |
| **Expert 2** |  |  |  |
| Evidence alignment | 4.240 ± 0.431 [4.120, 4.360] | 4.080 ± 0.274 [4.004, 4.156] | 4.020 ± 0.141 [3.981, 4.059] |
| Comprehensibility | 4.700 ± 0.505 [4.560, 4.840] | 4.660 ± 0.479 [4.527, 4.793] | 4.340 ± 0.479 [4.207, 4.473] |
| Relevance | 4.560 ± 0.705 [4.365, 4.755] | 4.500 ± 0.814 [4.274, 4.726] | 4.220 ± 0.932 [3.962, 4.478] |
| Empathy | 3.540 ± 0.734 [3.336, 3.744] | 3.120 ± 0.659 [2.937, 3.303] | 3.300 ± 0.544 [3.149, 3.451] |
| Feasibility | 4.300 ± 0.735 [4.096, 4.504] | 4.240 ± 0.822 [4.012, 4.468] | 3.800 ± 0.833 [3.569, 4.031] |
| **Expert 3** |  |  |  |
| Evidence alignment | 4.460 ± 0.503 [4.320, 4.600] | 4.160 ± 0.738 [3.955, 4.365] | 4.060 ± 0.512 [3.918, 4.202] |
| Comprehensibility | 4.940 ± 0.240 [4.874, 5.000] | 4.960 ± 0.198 [4.905, 5.000] | 4.960 ± 0.198 [4.905, 5.000] |
| Relevance | 4.060 ± 0.424 [3.942, 4.178] | 3.880 ± 0.659 [3.697, 4.063] | 3.880 ± 0.521 [3.736, 4.024] |
| Empathy | 3.480 ± 0.505 [3.340, 3.620] | 3.160 ± 0.584 [2.998, 3.322] | 3.140 ± 0.405 [3.028, 3.252] |
| Feasibility | 4.080 ± 0.340 [3.986, 4.174] | 3.980 ± 0.622 [3.807, 4.153] | 3.980 ± 0.428 [3.861, 4.099] |
