# Supplementary material for: A Supervised Fine-Tuned Large Language Model for Lifestyle Management in Patients With Prostate Cancer: Development and Evaluation Study
Source: J Med Internet Res. 2026 Jul 21;28:e92663. doi: 10.2196/92663 (PMC13387489; doi:10.2196/92663)
Supplement: Multimedia Appendix 10 [file jmir-v28-e92663-s010.docx]

**Multimedia Appendix 10. Full responses of the example QA pairs generated by PCaPLMM_SFT**

| **Scenario** | **Patient query** | **Model answer** |
| --- | --- | --- |
| Diet | How can I adjust my diet to better manage weight and maintain muscle strength while undergoing androgen deprivation therapy(ADT)? | Adjusting your diet during ADT is crucial for managing weight, maintaining muscle mass, and overall health. Here are some dietary adjustments you might consider:  1. Balanced Macronutrients:  - Carbohydrates: Aim for complex carbohydrates found in whole grains, fruits, and vegetables. These provide sustained energy and help maintain muscle mass.  - Proteins: Include lean proteins such as chicken, fish, beans, and legumes. These are essential for muscle maintenance and repair.  - Fats: Opt for healthy fats from sources like avocados, nuts, seeds, and olive oil. Limit saturated and trans fats.  2. Calcium and Vitamin D:  - Increase intake of calcium-rich foods (dairy, leafy greens, fish) and consider a supplement if necessary.  - Take a vitamin D3 supplement to ensure adequate absorption of calcium.  3. Hydration:  - Drink plenty of water throughout the day to stay hydrated and prevent dehydration, which can lead to muscle loss.  4. Limit Processed Foods:  - Reduce intake of processed foods, sugary drinks, and high-fat snacks. These can contribute to weight gain and negatively impact muscle mass.  5. Portion Control:  - Be mindful of portion sizes to avoid overeating. Use smaller plates and separate food groups to help control portions.  6. Regular Exercise:  - Combine dietary changes with regular physical activity, especially resistance training, to preserve muscle mass.  7. Consult a Professional:  - Work with a registered dietitian or nutritionist to tailor a diet plan that meets your specific needs and goals.  By making these dietary adjustments, you can better manage weight and maintain muscle strength during ADT. Always consult with your healthcare provider before making significant changes to your diet or exercise regimen. |
| Exercise | What types of exercise are recommended for prostate cancer patients at different stages? | Based on the available evidence, several types of exercise are recommended for prostate cancer patients at different stages:  1. Aerobic Exercise:  - Frequency: At least 30 minutes per session, three times per week.  - Intensity: Moderate intensity, where you can talk but not sing during the activity.  - Type: Activities such as walking, cycling, swimming, or using an elliptical machine are suitable options.  2. Resistance Exercise:  - Frequency: Twice per week.  - Intensity: Perform two sets of 8–15 repetitions at 60% of maximum exertion for large muscle groups.  - Type: Exercises involving weight lifting, resistance bands, or bodyweight exercises (e.g., push-ups).  3. Core Muscle Stabilization and Neuromotor Exercises:  - Frequency: Twice per week.  - Intensity: Moderate to high intensity.  - Type: Include exercises that focus on core stability and balance, such as standing on one leg or performing yoga poses.  4. Impact-Loading Exercises:  - Frequency: Once per week.  - Intensity: Perform 2–3 sessions per week, focusing on activities that create a small amount of impact.  - Type: Activities like jogging, running, or jumping on a box.  These recommendations are supported by various studies and guidelines, emphasizing the importance of regular physical activity in improving quality of life and reducing treatment-related side effects in prostate cancer patients. It is crucial for patients to consult with their healthcare provider before starting any new exercise regimen to ensure it is appropriate for their specific condition and treatment phase. |
| Psychological support | I’ve been feeling very anxious and stressed since being diagnosed with prostate cancer. What kind of psychological support can help me cope with this? | Prostate cancer patients often experience various forms of psychological distress, including stress, anxiety, depression, and emotional distress. These issues can significantly impact their quality of life and treatment outcomes. Here are some psychological supports that may help manage these challenges:  1. Psychological Support Groups: Joining a support group where individuals share similar experiences can provide emotional comfort and practical advice from others who understand what they're going through. These groups can be found locally or online, offering both face-to-face and virtual interactions.  2. Professional Counseling: Engaging with mental health professionals, such as psychologists or psychiatrists, can offer personalized strategies to cope with stress and anxiety. These professionals can help develop coping mechanisms and address any underlying psychological issues.  3. Social Support: Strong social networks play a crucial role in alleviating psychological distress. Family and friends can provide emotional support and practical assistance, helping the patient navigate their journey more effectively.  4. Educational Resources: Accessing educational materials about prostate cancer and its treatments can empower patients by providing them with knowledge about their condition and treatment options, reducing uncertainty and fear.  5. Religion and Spirituality: For some, religious practices or spiritual beliefs can serve as a source of strength and comfort, helping them maintain a positive outlook during difficult times.  6. Self-Efficacy Building: Increasing self-efficacy can empower patients to take control over their lives and manage their health better. This involves building confidence in one's ability to handle challenges and achieve goals.  7. Cognitive Behavioral Therapy (CBT): CBT is a structured form of therapy that helps patients change negative thought patterns and develop healthier coping mechanisms. It has been shown to be effective in managing anxiety and depression in cancer patients.  8. Mindfulness and Relaxation Techniques: Practices such as meditation, yoga, and deep breathing exercises can help reduce stress and improve overall well-being.  9. Online Support Platforms: Platforms like the CHESS Interactive Online Support system have been utilized to provide ongoing support to newly diagnosed prostate cancer patients, addressing their information needs and improving their quality of life.  Each patient's needs are unique, so it is essential to explore different types of support and find what works best for them personally. |
| Medication adherence | If I miss a dose or don’t take my prostate cancer meds on time, is that a big deal? What should I be worried about? | Adherence to prescribed medications plays a crucial role in managing various health conditions effectively. Non-adherence can lead to several negative outcomes:  1. Reduced Treatment Efficacy: Not following your medication schedule as prescribed can decrease the effectiveness of your treatment, potentially leading to disease progression or recurrence.  2. Worsened Survival Rates: In cancer management, non-adherence can negatively impact survival rates by allowing the disease to progress unchecked.  3. Development of Resistance: Inconsistent use of certain medications can contribute to the development of drug-resistant pathogens, making future treatments less effective.  4. Increased Health Care Costs: Non-adherence often requires additional medical interventions due to complications, which can lead to higher healthcare costs.  5. Impact on Quality of Life: Poor adherence can lead to more frequent hospital visits, increased discomfort, and reduced ability to perform daily activities, negatively affecting overall quality of life.  To ensure optimal treatment outcomes, it's essential to follow your prescribed medication regimen closely. If you encounter any challenges with adherence—such as side effects, difficulty remembering doses, or financial concerns—it's important to discuss these issues with your healthcare provider. They may offer solutions such as adjusting dosages, providing reminders, or offering support groups to help maintain consistency in your treatment plan. |
| Weight management | As a prostate cancer patient, I’m concerned that my current weight might increase my risk of heart problems. What are some safe ways for me to manage my BMI? | To safely manage your BMI and reduce the risk of cardiovascular issues, consider adopting a balanced approach that includes dietary modifications, regular physical activity, and maintaining a healthy lifestyle. Here are some specific recommendations:  1. Dietary Modifications:  - Balanced Diet: Focus on consuming a diet rich in fruits, vegetables, whole grains, lean proteins, and healthy fats. Limit processed foods, sugary beverages, and high-calorie snacks.  - Portion Control: Be mindful of portion sizes to avoid overeating.  - Hydration: Drink plenty of water throughout the day to stay hydrated.  2. Physical Activity:  - Regular Exercise: Aim for at least 150 minutes of moderate-intensity aerobic activity or 75 minutes of vigorous-intensity activity each week, along with muscle-strengthening activities on two or more days a week.  - Strength Training: Incorporate strength training exercises to build muscle mass, which can help increase metabolism.  3. Weight Management:  - Monitor Weight: Regularly monitor your weight to ensure it remains within a healthy range.  - Avoid Unhealthy Weight Loss: Avoid rapid weight loss, as it might lead to muscle loss and increase the risk of osteoporosis.  4. Lifestyle Changes:  - Sleep: Ensure you get enough sleep, as poor sleep can negatively impact heart health.  - Stress Management: Practice stress-reduction techniques such as meditation, yoga, or deep breathing exercises.  5. Consultation with Healthcare Providers:  - Professional Guidance: Regular consultations with healthcare providers can provide personalized advice and monitoring.  By integrating these lifestyle changes, you can effectively manage your BMI and reduce the risk of cardiovascular issues. Always consult with your healthcare provider before making significant changes to your diet or exercise regimen. |
